# Wafer‐Scale Manufacturing and Crack‐Free Transferring of GaN‐Based Membranes for Flexible Optoelectronics

**DOI:** 10.1002/advs.202512193

**Published:** 2025-09-23

**Authors:** Yaqi Gao, Kaixuan Zhou, Zhetong Liu, Lulu Wang, Yiwei Duo, Shenyuan Yang, Jiankun Yang, Xiang Gao, Wenze Wei, Junxi Wang, Peng Gao, Jinmin Li, Zhongfan Liu, Jingyu Sun, Tongbo Wei

**Affiliations:** ^1^ Research and Development Center for Wide Bandgap Semiconductors, Institute of Semiconductors Chinese Academy of Sciences Beijing 100083 China; ^2^ Center of Materials Science and Optoelectronics Engineering University of Chinese Academy of Sciences Beijing 100049 China; ^3^ Beijing Huairou Laboratory Beijing 101400 China; ^4^ College of Energy, Soochow Institute for Energy and Materials Innovations, Jiangsu Provincial Key Laboratory for Advanced Carbon Materials and Wearable Energy Technologies Soochow University Suzhou 215006 China; ^5^ Beijing Graphene Institute (BGI) Beijing 100095 China; ^6^ Center for Nanochemistry (CNC), Beijing Science and Engineering Center for Nanocarbons, Beijing National Laboratory for Molecular Sciences, College of Chemistry and Molecular Engineering Peking University Beijing 100871 China; ^7^ Electron Microscopy Laboratory, and International Center for Quantum Materials, School of Physics Peking University Beijing 100871 China; ^8^ State Key Laboratory of Superlattices and Microstructures, Institute of Semiconductors Chinese Academy of Sciences Beijing 100083 China

**Keywords:** flexible LEDs, flexible PDs, GaN, graphene, Si(100)

## Abstract

Heterogeneously integrated devices have great demand for the freestanding wafer‐level wide bandgap semiconductors in their single crystal membrane form. However, the current fabrication strategy is quite costly and low throughput. Here, quasi van der Waals epitaxy (QvdWE) of nearly single crystalline GaN membranes on Si(100) substrate via directly‐grown graphene interfacial layers are demonstrated. Through simple chemical etching, wafer‐scale III‐nitride membranes can be readily realized and transferred onto arbitrary substrates, with minimized damage and wafer‐scale peeling capability. The obtained flexible InGaN‐based light emitting diode device demonstrates strong blue luminescence due to avoiding the crack during transfer process. Meanwhile, flexible ultraviolet photodetector also shows good stability, delivering high responsivity and specific detectivity of 3.52×10^4^ A/W and 6.21×10^12^ Jones, respectively. This work indicates that the QvdWE heteroepitaxy and intact transfer strategies can help the combination of GaN‐based devices and Si‐based integrated circuits.

## Graphical abstract

QvdWE growth of III‐V nitrides on Gr/SiO_2_ not only helps to guide unified crystal orientation of GaN and improve crystalline quality, but also allows wafer‐level damage‐free GaN membrane to be easily transferred to foreign substrates through chemical etching of SiO_2_. The flexible vertical light‐emitting diode and ultraviolet photodetector devices are fabricated and compared.

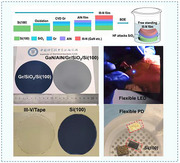

## 1 Introduction

3D heterogeneous integration has become a key technology for achieving multi‐functional semiconductor production, including display devices and human‐computer interaction devices, especially the flexible and wearable devices area.^[^ ^1^, ^2^, ^3^, ^4^ ^]^ By stacking various types of III‐V nitride membranes with Si platform, heterogeneous integration promises to solve the shortage of Si‐based light sources and take advantage of the natural characteristics of direct adjustable bandgap and high wavelength selectivity for III‐V group materials.^[^ ^5^, ^6^ ^]^ At present, the transfer of III‐V nitride membranes and devices has generally been performed using the following main strategies: chemical lift‐off (CLO),^[^ ^7^ ^]^ laser lift‐off (LLO),^[^ ^8^, ^9^ ^]^ and van der Waals (vdW) transfer technology using 2D materials.^[^ ^10^, ^11^, ^12^ ^]^ Various CLO methods have been demonstrated for substrate removal, including direct corrosion of the Si substrate. However, how to maintain the integrity of the released thin‐film in the liquid poses another problem, necessitating the protection of metal electrodes and the avoidance of by‐product adhesion.^[^ ^13^ ^]^ Currently, relatively mature LLO technology not only requires a complex process and high cost, but also inevitably causes some damage to the interface of the peeling layer, therefore requiring sufficient epilayer thickness to protect the upper multiple quantum wells (MQWs) structure.^[^ ^14^ ^]^

Compared to the first two methods mentioned above, the recently reported quasi van der Waals epitaxy (qvdWE)^[^ ^15^, ^16^ ^]^ or remote epitaxy (RE)^[^ ^17^, ^18^ ^]^ through 2D materials to grow III‐nitrides provides a simple pathway for the mechanical exfoliation of epilayer structure. With the introduction of 2D materials, it is possible to guide the orientations of wurtzite nitride and reduce the high strain and dislocation density caused by large lattice and thermal mismatch.^[^ ^19^ ^]^ The weak interlayer coupling of 2D materials may allow the membrane exfoliation for heterogeneous integration and flexible devices using mechanical force.^[^ ^20^, ^21^, ^22^ ^]^ Low‐damage III‐V thin membranes can be obtained with controllable thickness and a smooth separated interface. However, to provide enough dangling bonds to nucleate on 2D materials, plasma treatment or NH_3_ treatment are usually introduced onto either graphene (Gr) or hexagonal boron nitride (h‐BN),^[^ ^23^, ^24^, ^25^ ^]^ and thus it is easy to unintentionally destroy the 2D material and cause hole epitaxy or even peel failure. Furthermore, it is challenging to preserve suitable adhesion without spontaneously delaminating and simultaneously avoiding the introduction of cracks during the mechanical exfoliation of nitride.^[^ ^26^, ^27^ ^]^ Low mass production and uncontrollable cracks introduction have become the bottlenecks restricting the development of flexible devices and 3D integration.^[^ ^28^, ^29^ ^]^ Therefore, to achieve controllable peeling with a highly repeatable method becomes highly imperative. The dual function of transfer‐free 2D materials for high‐quality GaN and peelable membrane production is hoped to be utilized.^[^ ^30^ ^]^

In this work, based on the combination of natural SiO_2_ and few‐layer Gr as a buffer layer, we have ensured wafer‐level lossless stripping of GaN membrane by chemical etching. We directly grow continuous Gr on SiO_2_/Si(100) substrate and then realize direct QvdWE growth of nearly single‐crystalline GaN film on the amorphous substrate. QvdWE growth on Gr/SiO_2_ not only helps to guide unified crystalline orientation of GaN and improve crystalline quality, but also allows wafer‐level damage‐free GaN membranes to be easily transferred to foreign substrates through chemical etching of SiO_2_, recycling the Si(100) substrate to reuse. We also fabricate the flexible vertical light‐emitting diode (LED) and ultraviolet photodetector (UV PD) devices without cracks. These results hold great potential in the integration of III‐V devices with the Si platform and 3D integration.

## 2 Results and Discussion

**Figure 1:**
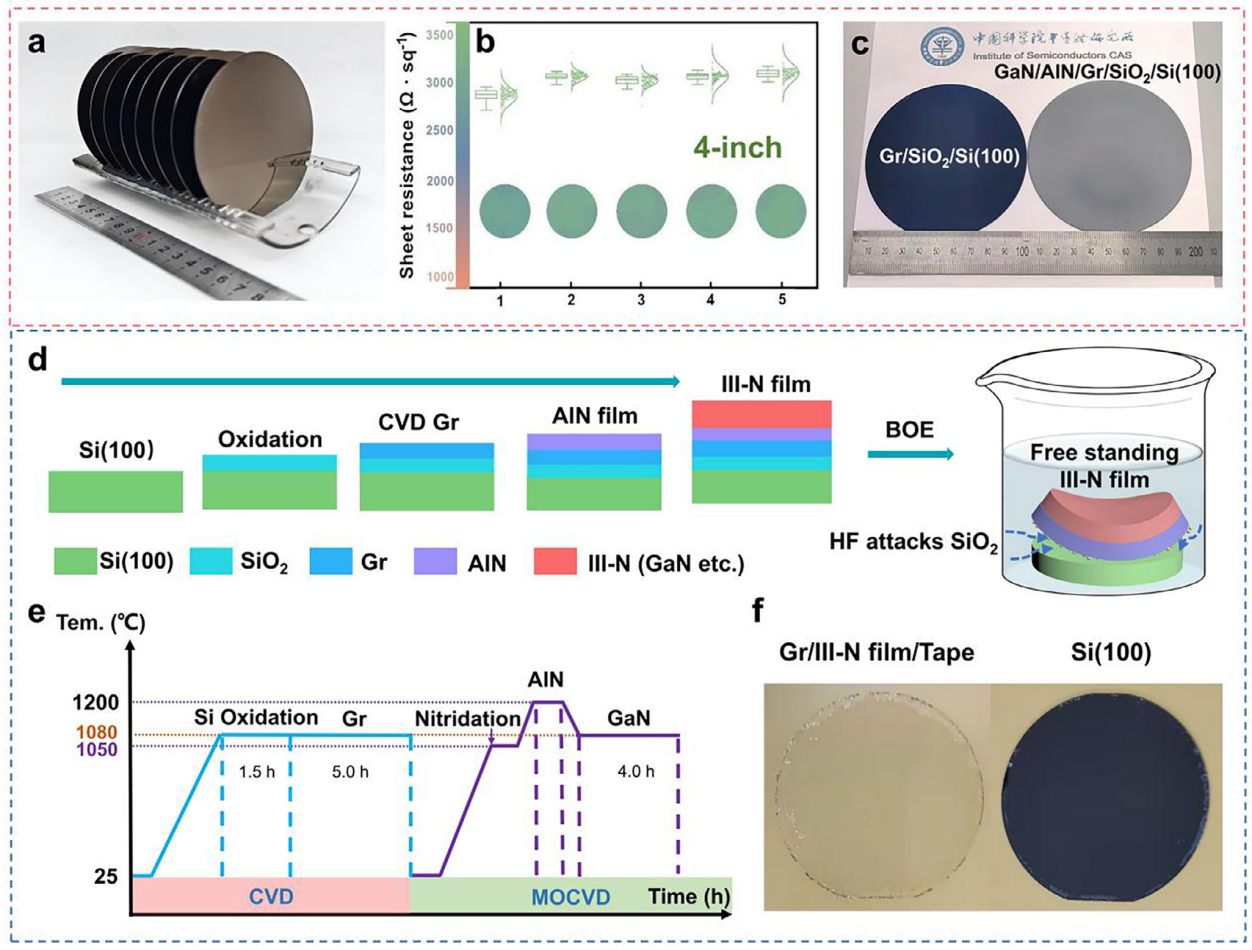
Epitaxial growth and exfoliation processes of wafer‐level III‐nitride membranes on Si(100) substrates. a) Photograph of 4‐inch Gr wafers; b) Sheet resistance maps of the 4‐inch Gr/SiO_2_/Si(100) wafers; c) Photographs of 4‐inch GaN wafer on Gr/SiO_2_/Si(100) substrate; d) The schematic preparation process and stripping method of III‐nitride film on Gr/SiO_2_/Si(100); e) Growth process of III‐V GaN film on Si(100) substrate; f) Photograph of III‐N GaN membrane after stripping.

A continuous Gr film is directly grown on SiO_2_/Si(100) substrate by low‐pressure chemical vapor deposition (LPCVD) at a synthetic temperature of 1050 °C. With this method, 4‐inch Gr/SiO_2_/Si(100) wafers could be prepared in large quantities as shown in **Figure** **1**a. To assess the electrical uniformity of the 4‐inch Gr/SiO_2_/Si(100) wafers, sheet resistance (Rs) is mapped using four‐probe resistance measurements. The average Rs value is 3000 Ω sq^−1^ accompanied by a narrow distribution in Figure 1b. Microscopic images and Raman spectra results both confirm the uniformity of the Gr/SiO_2_/Si(100) wafer (Figures S1 and S2, Supporting Information). Based on the position of the G peak in the Raman spectra results, disregarding the influences of temperature, doping and minor strain on the sample, the layer number of Gr is estimated to be 2.^[^ ^31^ ^]^ Based on these wafers, 4‐inch GaN films are obtained using metal organic chemical vaper deposition (MOCVD). Figure 1c exhibits a macroscopically homogeneous GaN/Gr/SiO_2_/Si(100) wafer, as indicated by its identical color reflection. The scanning electron microscopy (SEM) image and energy dispersive spectroscopy (EDS) spectrum of cross‐section of GaN film are shown in Figure S3 (Supporting Information). Through multiple processes in Figure 1d, we obtain large‐size III‐nitride films on Si(100) substrates. The preparation conditions for the details are shown in Figure 1e. Meanwhile, the flexible III‐group GaN membrane could be rapidly prepared by chemical etching of SiO_2_ layer in HF or buffered oxide etchant (BOE). Photographs of III‐group GaN membranes and Si(100) substrates after peeling are shown in Figure 1f. Unlike the conventional mechanical exfoliation, these results demonstrate the feasibility of the preparation of wafer‐level flexible III‐nitrides films without introducing the cracks. The Si(100) substrate can be recycled, which enables low‐cost fabrication of Si‐based optoelectronic devices and flexible III‐nitride devices.

The poor crystalline quality of Si‐based III‐V group nitride has been limiting its development. On this issue, we first obtain high‐quality GaN films using the QvdWE method. The key role of AlN nucleation layer in improving crystalline quality is analyzed. The schematic process of growing GaN film on Gr‐coated SiO_2_/Si(100) is shown in Figure S4a—f (Supporting Information). If the epitaxy of GaN epilayer is carried out directly on the surface of the Si(100) substrate, the melt‐back effect will occur due to the reaction between Si and Ga. As a common approach, the AlN buffer layer not only effectively suppresses Si diffusion, but also provides a large compressive strain for the subsequent GaN epitaxial layer, avoiding the cracks in the GaN film.^[^ ^32^, ^33^ ^]^ Through a variety of growth comparisons, it can be seen that both the Gr/SiO_2_ layer and the AlN buffer layer are indispensable (Figure S5a—c, Supporting Information). Meanwhile, these results also reveal the transfer‐free Gr/SiO_2_ could be utilized as a dual function for high‐quality GaN and peelable membrane production.

**Figure 2:**
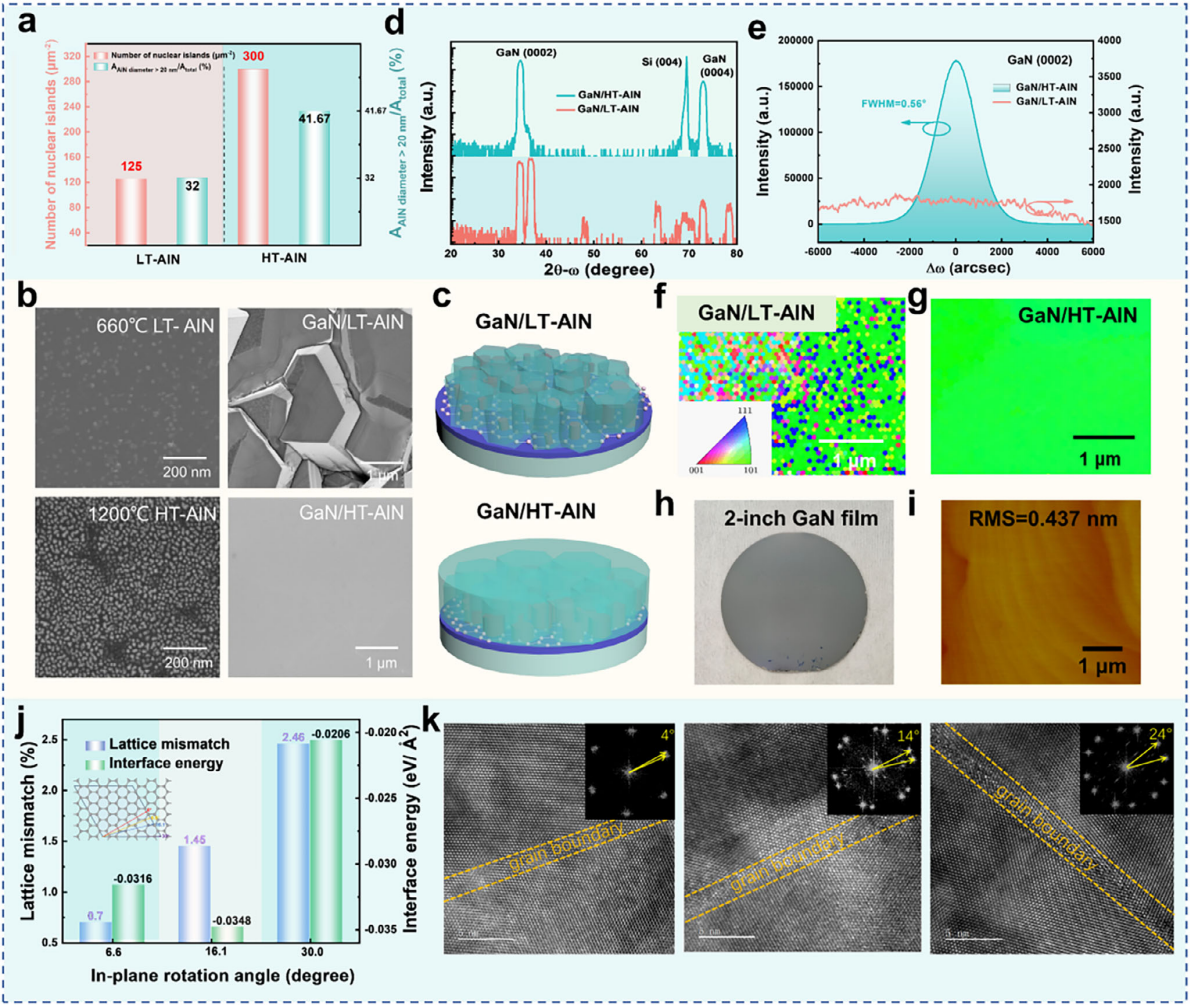
Epitaxial growth of AlN buffer layers and GaN films on Gr/SiO_2_/Si(100) substrate. a) Statistics of AlN nucleation islands grown on SiO_2_/Si(100) at 660°C and 1200°C; b) SEM images of AlN buffer layers and GaN films; c) Schematic of out‐of‐plane orientation of GaN and AlN islands; d) XRD 2θ‐ω scan curves of GaN/LT‐AlN/Gr/SiO_2_/Si(100) and GaN/HT‐AlN/Gr/SiO_2_/Si(100); e) X‐ray rocking curves of GaN/LT‐AlN/Gr/SiO_2_/Si(100) and GaN/HT‐AlN/Gr/SiO_2_/Si (100); Corresponding out‐of‐plane EBSD maps of GaN films with LT‐AlN f) and HT‐AlN g); Photograph h) and AFM image i) of a 2‐inch GaN wafer; j) The interaction energies and lattice mismatch of three relatively dominant epitaxial relationships for GaN/Gr system; k) The various grain boundaries between adjacent GaN grains. The insets show FFT images from the boundary with the relative rotation angles.

To increase the surface activity of Gr and generate more nitride nucleation islands,^[^ ^34^ ^]^ NH_3_ pre‐treatment of Gr is employed in the MOCVD growth chamber. Then, the AlN nucleation layers with different growth temperature of 660 °C and 1200 °C are grown on the Gr surface, following by the epitaxial growth of GaN film. Nucleation statistics and SEM images are shown in **Figure** **2**a,b. The AlN buffer layer grown at lower temperature (LT‐AlN) demonstrates low nucleation density and small nucleation size, while the AlN buffer layer grown at high temperature (HT‐AlN) shows high nucleation density and large size of nuclear islands. The difference in initial AlN nucleation density and island sizes leads to the different morphology of the GaN epitaxial layer (Figure 2b).

The adsorption and diffusion mechanisms of Al adsorbed atoms on the substrate are closely related to the initial growth mode of AlN, which could be analyzed using an Arrhenius‐type exponential law^[^ ^35^ ^]^

(1) $$r=\mathrm{vexp}\left(-E_{\mathrm{diff}}/\mathrm{kT}\right)$$

where *r*, *v*, *E_diff_*, *k* and *T* are the diffusion rate, the attempt frequency, diffusion barrier, the Boltzmann constant and the temperature, respectively. When controlling the same pre‐treatment conditions for Gr and the same flow‐rate parameters, the growth temperature of the AlN nucleation layer affects the surface morphology of the epitaxial layer by changing the initial nucleation density and size of the AlN nucleation islands. If the temperature is too low, the atomic clusters can't obtain enough energy to overcome the diffusion barrier on the substrate surface. Meanwhile, the insufficient decomposition of the precursor also leads to the sparsity of nuclear islands as shown in the Figure 2b. In contrast, HT‐AlN nucleation layer ensures uniform adsorption and diffusion, and the atomic clusters rapidly coalesce to reduce interface energy, increasing the nucleation center's size. The strong adsorption between Al atoms on defect sites of Gr, as well as the low diffusion barrier and free diffusion in the region without defects, enable the effective nucleation, high crystalline quality and rapid lateral growth of the AlN buffer layer on Gr. As shown in Figure 2c, the high density and guided orientation of the AlN nucleation layer directly affect the quality of the subsequent GaN epilayer.

Furthermore, high‐resolution X‐ray diffraction (HRXRD) analysis is used to evaluate the crystalline quality of the as‐grown GaN films. Only the peaks corresponding to GaN (0002) and Si (004) can be observed in Figure 2d, indicating the GaN with HT‐AlN buffer layer has the uniform c‐axis orientation. On the contrary, GaN film with LT‐AlN buffer shows the polycrystalline features, presenting multiple diffraction peaks. These results are consistent with optical microscope images (Figure S5d, e, Supporting Information). X‐ray rocking curve full width at the half maximum (FWHM) of the (0002) peak of GaN film on HT‐AlN/Gr/SiO_2_/Si(100) is 0.56° in Figure 2e, demonstrating the much improved GaN quality as compared to previous GaN results on amorphous substrate.^[^ ^36^, ^37^, ^38^, ^39^, ^40^, ^41^, ^42^, ^43^, ^44^, ^45^ ^]^ The Raman results show that the as‐grown GaN films are close to strain‐free, and the Gr layers still exist after suffering from high temperature (Figure S5f, Supporting Information). The role of HT‐AlN nucleation layer is also proven in EBSD images, which can provide the driving force to grow the c‐axis orientation of GaN epilayer on Gr (Figure 2f,g; Figure S6a, b, Supporting Information). Figure 2h indicates that there is no severe melt‐back effect according to our growth method. The atomic force microscopy (AFM) image of GaN film shows a typical atomic step flow morphology, with the root mean square (RMS) roughness of only 0.437 nm in scanned area of 5×5 µm2 in Figure 2i.

Due to the amorphous nature of the SiO_2_ layer on Si(100), the orientation of Gr dominates the orientation of subsequent grown nitrides. Within a single Gr domain, AlN islands with the dangling bonds of Gr uniformly follow the in‐plane orientation of single‐crystalline Gr. However, the wafer‐scale Gr is spliced together by the many domain regions, directly affecting the crystallographic direction of nitrides, especially for the in‐plane orientation. The in‐plane orientation is investigated by density functional theory (DFT) method. Three relatively dominant lattice‐matched epitaxial relationships for III‐nitrides/Gr system are shown in Figure 2j and Figure S7 (Supporting Information). The GaN grains with various in‐plane orientations on Gr/SiO_2_/Si(100) substrate will form a relative misorientation. High‐resolution transmission electron microscopy (HRTEM) and corresponding fast Fourier transform (FFT) images show the deviation of plane angles between boundaries of adjacent GaN domains to analyze grain boundary characteristics of in‐plane oriented GaN. The overlap of two GaN domains with a relative misorientation causes a form of a clear Moore pattern. As shown in Figure 2k, the in‐plane rotation angles are 4°, 14°, and 24°, respectively. We obtain nearly single‐crystalline GaN film with a highly c‐axis‐oriented crystalline structure. These results are well matched to studies of Liu's group. Liu et al. showed the GaN grown on glass substrate had multiple preferred relative angles of in‐plane oriented domains, such as 0°, 3°, 13.9°, 23.9°, 19°, and 29°.^[^ ^41^, ^43^ ^]^ These results indicate that there are the nitride domains with relative angular, which is close to the nearly lattice‐matched epitaxial relationships for GaN/Gr alignment. The in‐plane orientation of each AlN domain is aligned by oriented attachment under high temperature on slippery Gr surface, which results in nearly single‐crystalline of GaN epilayer.

**Figure 3:**
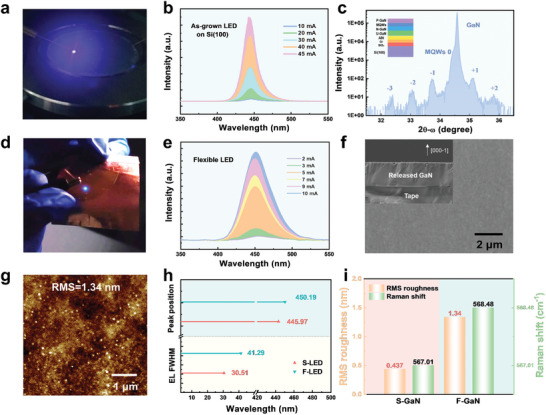
Characteristics of GaN‐based LED before and after stripping. a) Photograph of the blue EL emission of the LED on Si(100) substrate; b) EL spectra of LED; c) XRD 2θ‐ω scan of LED showing the good periodicity and high quality of MQWs; d) Photograph of the blue EL emission of the flexible LED; e) Room temperature EL spectra of the flexible LED at various currents, which show a peak wavelength of ≈450 nm; f) SEM image of backside nitride exposed after BOE etching of LED epitaxial film with HT‐AlN buffer layer, the inset is the cross‐sectional image; g) AFM image of backside nitride surface after stripping; h) Comparison of peak position and FWHM of EL spectra at 7 mA for S‐LED (before stripping and F‐LED (after stripping); i) Comparison of RMS roughness values and Raman shifts for S‐GaN film (before stripping) and F‐GaN film (after stripping).

The flat and uniform surface morphology with a high c‐axis orientation of the obtained GaN film enables to fabricate LEDs on Gr‐coated SiO_2_/Si(100) substrate. The LEDs structure is shown in Figure S8a (Supporting Information), which is composed of a u‐GaN layer, an n‐GaN layer, five‐period In_x_Ga_1–x_N/GaN MQWs, and a p‐GaN layer. Each pair InGaN/GaN MQW of LED with 3‐nm‐thick well and 12‐nm‐thick GaN barrier layers are grown at 740 °C/810 °C, respectively. Figure S8b (Supporting Information) shows the uniform, smooth surface of 2‐inch LED wafer. The as‐grown LED wafer emits the strong blue light emission in **Figure** **3**a. The good luminescent properties are demonstrated according to electroluminescence (EL) spectra with a range of 10–45 mA in Figure 3b. The as‐grown LED shows a peak position around 445 nm. The XRD 2θ‐ω scan profile demonstrates the strong GaN (0002) peak and multiple satellite peaks from the MQWs up to the third order, indicating good periodicity and high quality of MQWs in Figure 3c.

To verify the optical characteristics of blue LEDs, the temperature dependence photoluminescence (PL) spectra are measured as shown in Figure S8c—e (Supporting Information). Usually, the ratio of the PL intensities of 300 K and 10 K are widely used to evaluate the internal quantum efficiency (IQE) [η_PL_ = 100%×(I_300K_/I_10K_)].^[^ ^46^, ^47^, ^48^ ^]^ Here, IQE of the blue LED grown on Gr/SiO_2_/Si(100) is about 11.47%. The current‐voltage (I‐V) curve is shown in Figure S8f (Supporting Information). As shown in the Figure 3d, the wafer‐scale GaN‐based LED membrane could be fabricated. The etching of SiO_2_ layer could also be easily realized using chemical reagents of HF after the completion of the metal electrode production. The Ni/Au electrode is not easily damaged by BOE solution, while the Ti/Al/Ti/Au electrode is easily dissolved, thus deteriorating the performance of devices. Therefore, we evaporate Ni/Au stacks on the LED epilayer as the bottom electrode for flexible vertical structure LED. The fabrication process of vertical structure LED is simple as shown in Figure S9a—c (Supporting Information), which may promote heat dissipation and luminous efficiency.^[^ ^49^, ^50^ ^]^ LED membrane is transferred onto the foreign substrate, such as Cu foil in Figure 3d. In the bent state, the vertical structure LED still displays bright blue luminescence, indicating the good performance of flexible LED. The EL intensity gradually increases with the current in Figure 3e. SEM images of the vertical structure LED membrane have been shown in Figure 3f, with a smooth and low‐damage surface on a wafer scale (Figure S9d, Supporting Information). The AFM image of N‐polarity backside after removing SiO_2_ shows a low RMS roughness value of 1.34 nm (Figure 3g). On the other hand, Figure S9e (Supporting Information) shows a rougher surface than Figure 3f, which is due to the poorer quality of GaN/AlN film. The higher the quality of the GaN film obtained, the smoother it is after exfoliation, which also illustrates the importance of the nucleation layer. Unlike the front surface, the release surface is the N‐polarity face and can be easily etched by KOH solution to form rough morphology (Figure S9f, Supporting Information). The difference of EL for as‐grown LED on Si(100) and flexible LED are shown in Figure 3h. The original LED shows a FWHM of 30.51 nm at 7 mA and a peak position of 445.97 nm before the exfoliation, while the FWHM of flexible LED membrane broadens to 41.29 nm and the peak red‐shifts to 450.19 nm. The variation curves of peak position and FWHM are shown in the Figure S10 (Supporting Information). The difference in LED performance may originate from the change in membrane strain, metal‐semiconductor contact, and roughed surface. The evaluation of the performance changes of devices and components is also a key issue that needs to be addressed for the widespread application of III‐V nitride flexible devices. Applying quantifiable stress or repeatable bending tests helps identify the causes and lays the foundation for the large‐scale application of flexible devices.

For transferred film, the surface roughness of N‐polarity backside will increase inevitably, as shown in Figure 3i. The Raman spectrum is also analyzed to determine the strain distribution in the GaN membrane. Raman spectrum shows that Gr signals can only be found on the GaN membrane but not on the Si(100) substrate, thus indicating that the exfoliation process occurs between the SiO_2_ layer and Gr (Figure S1a, b, Supporting Information). The E_2_ (high) mode phonon frequency of strain‐free GaN is located at 568 cm^−1^ according to the reported value.^[^ ^51^ ^]^ After the exfoliation, the Raman peak of GaN shifts from 567.01 to 568.48 cm^−1^, indicating that the tensile strain of GaN is released and even turns into compressive strain on the target substrate (Figure 3i). Hence, following the increase of the compressive strain in InGaN MQWs, the polarization electric field is strengthened. The intensification of the quantum confinement Stark effect (QCSE) leads to the broadening of FWHM of the emission spectra and the red shift of the luminescence wavelength.^[^ ^52^, ^53^ ^]^ On the other hand, the large red shift of the wavelength is also caused by the reduction of the band gap of GaN due to the increase of junction temperature after removing the Si substrate.^[^ ^54^ ^]^ And another possibility of FWHM broadening is because the current stripped support layer is a tape as an adhesive layer. When the force from the probe is applied to the tape, the adhesive layer is prone to deformation or even breakage,^[^ ^29^ ^]^ which may affect the contact between the probe and the electrode and form microcracks that are difficult to observe. This problem is also being continuously optimized to explore new foreign substrates that are conductive, adherent, and elastic. The another innovation is to allow the device to be released and translated onto any substrate without inverting the front surface. We optimized the transfer method of the PD with a transverse conductive structure using polymethyl methacrylate (PMMA), which can effectively avoid this problem.

**Figure 4:**
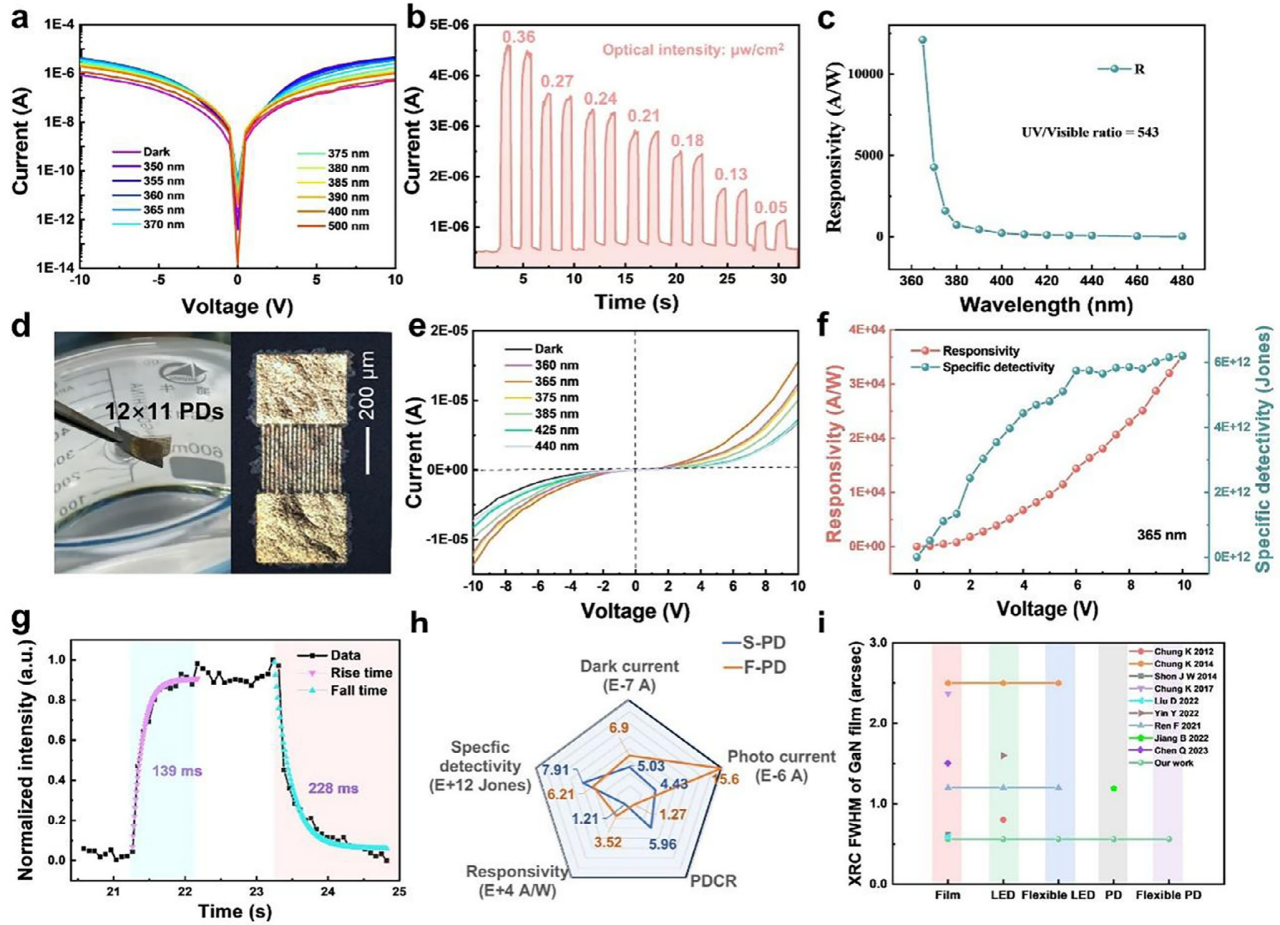
Characterizations of MSM GaN PD before and after stripping. a) I‐V curves of as‐grown PD at various illuminations; b) Optical intensity‐related current curve; c) Responsivity as a function of the wavelength; d) The PD arrays were peeled off and a PD was transferred to glass; e) I‐V curves of flexible PD; f) The responsivity and the specific detectivity at various voltages under 365 nm UV illumination; g) The time response curve and its exponential fitting curves for flexible PD to obtain the rise time and fall time;h) Comparison of performance at 10 V for S‐PD (before stripping) and F‐PD (after stripping); i) A summary of GaN‐based materials and devices on amorphous substrates in recent years.^[^ ^34^, ^36^, ^37^, ^38^, ^39^, ^40^, ^41^, ^42^, ^43^ ^]^

Meanwhile, we directly use GaN epilayer to prepare transverse conductive metal‐semiconductor‐metal (MSM) structure UV PDs. I‐V curves at various wavelengths are shown in **Figure** **4**a. The maximum photocurrent is obtained around the 365 nm UV illumination, which is related to the band gap of GaN. As the optical power density changes, the response is sensitive and repeatable (Figure 4b). The UV/Visible ratio is 543 according to the responsivities of 365 nm and 480 nm (Figure 4c). Based on the above excellent performance of Si‐based MSM PD, flexible PDs are also prepared as shown in the Figure S12 (Supporting Information). A layer of PMMA is used to reduce the damage of PD after wet‐etching. After coating and curing of PMMA on the surface of PD, free‐standing GaN PD membrane can be soaked in BOE solution to etch the SiO_2_ layer, allowing the PD to exfoliate from the Si(100) substrate and float in the solution (Figure 4d left). Subsequently, PMMA can be dissolved in acetone, enabling the PD arrays with low damage as shown in Figure S13 (Supporting Information). A flexible MSM GaN UV PD transferred to glass with transverse conductive interdigital structure is shown in Figure 4d, right. They also can be transferred to any substrate, as shown in Figure S14 (Supporting Information). The appropriate etching time for coating different substances on the surface of GaN films has also been studied, as shown in the Figure S15a (Supporting Information). The excellent performance of the PD remains good during the entire transfer process (Figure S15b, Supporting Information).

The GaN‐based flexible PD shows a sensitive optical response with a low dark current level of µA at 10 V in Figure 4e. The responsivity and the specific detectivity increase with the increase of the applied bias, while the specific detectivity tends to saturate, possibly due to the dark current increasing significantly with voltage (Figure 4f). At 10 V, the responsivity and the specific detectivity are 3.52×10^4^ A/W and 6.21×10^12^ Jones, respectively. The spectral measurement shows that the cutoff edge of flexible GaN PD is below 380 nm (Figure S16a, Supporting Information). The UV/Visible ratio is 3176, which is 5.85 times that of original PD. Figure S16b (Supporting Information) also shows the time‐resolved photocurrent of flexible PD in response to the turn‐on and turn‐off of 365 nm UV illumination, which demonstrates the strong capability of detecting the UV signal. In multiple 2 s on/off tests using the ordinary xenon lamp, the rise time and fall time are 139 ms and 228 ms, respectively (Figure 4g). Figure S17 (Supporting Information) shows the fitting curves of response time for flexible GaN PD by single exponential equation.^[^ ^55^ ^]^ The comparison of key parameters of the PD before stripping (S‐PD) and after stripping (F‐PD) is shown in the Figure 4h, indicating that the flexible PD has good sensitivity and meets practical requirements.

In addition, the vertical conductive structure PD is also fabricated using a similar process with vertical structure LED and the I‐V curves are shown in the Figure S18 (Supporting Information). The responsivity is 0.9 mA W^−1^ and the specific detectivity is 5.39×10^7^ Jones. The low dark current and responsivity of the vertical conductive structure PD may originate from the insulation of the AlN buffer layer, which leads to a decrease in the gain of PD.^[^ ^56^, ^57^ ^]^ A summary of GaN‐based materials and devices on amorphous substrates is displayed in Figure 4i and Table S1 (Supporting Information). Therefore, we successfully achieve wafer‐scale high‐quality GaN membranes without any cracks, simplifying the process of epitaxy and device integration and realizing the functions of Si‐based light source and photodetection.^[^ ^58^ ^]^ And flexible LEDs and PDs are obtained using wet etching, expanding the application scenarios of III‐group nitrides. Our results comprehensively demonstrate the crystalline quality and optoelectronic device performance of GaN‐based nitrides, which have obvious advantages and application prospects compared to recent reports, providing a significant reference for subsequent research.

As the wafer area expands, the epitaxial difference from the center to the edge is also amplified. Within‐wafer uniformity of films and devices needs to be considered to avoid the generation of edge defects caused by edge stress and temperature differences, which may affect the yield of devices. These put forward high requirements for the airflow optimization and temperature control of MOCVD. But compared to other substrates, the preparation of large‐area Si substrates and Gr has laid the foundation for the epitaxy and transfer of III‐V nitrides. Based on the above exploration, it is hoped that the preparation and transfer of large‐area III‐V nitrides can be realized with the help of mature Si wafer manufacturing.

## 3 Conclusion

By directly‐grown Gr grown on CMOS‐compatible SiO_2_/Si(100) substrate, we obtain the wafer‐scale GaN membrane using the simple chemical etching. The HT‐AlN buffer on Gr enables nearly single‐crystalline GaN epilayer with a highly c‐axis‐oriented crystalline structure, rendering markedly improved GaN quality as compared to previous reports. The flexible vertical LED with strong blue luminescence is presented and the fast response and reliability of the flexible PD are ensured via fast switching. After overcoming the challenges of III‐V group light sources and Si‐based heterojunction epitaxy, the functional nitride material system will help to accelerate the development of hybrid integration and flexible application for the new generation of inorganic optoelectronic devices.

## 4 Experimental Section

### CVD Growth of Gr on SiO_2_/Si(100) Substrate

Gr growth on a Si wafer was performed using a LPCVD system. A commercially available 2‐inch Si(100) substrate was subjected to a cleaning process involving deionized water and ethanol. Following cleaning, the Si wafer was loaded into a three‐zone high‐temperature furnace. The furnace was heated to a target temperature of 1080 °C and allowed to stabilize for approximately 10 min. During this stabilization period, a controlled gas flow of 800 sccm of argon (Ar) and 500 sccm of hydrogen (H_2_) was established within the furnace. This gas mixture created a reducing atmosphere conducive to Gr growth and helped to maintain a stable growth environment. To introduce the carbon source required for Gr synthesis, ethanol was introduced into the reaction chamber at a controlled flow rate of 0.48 L min^−1^. The growth process was carried out for a duration of 4 hours under a pressure of 1000 Pa.

### MOCVD Growth of GaN‐Based Devices on Gr/SiO_2_/Si (100) Substrate

The CVD Gr/SiO_2_/Si(100) substrate was first pretreated with an in situ NH_3_ flow in the Veeco K300 MOCVD chamber at ≈1100°C. Trimethylgallium (TMGa), trimethylaluminum (TMAl), trimethylindium, and NH_3_ were employed as the reactants for growing nitride films. The AlN buffer layer was grown for 2 min at temperature of 700 °C and 1200°C, respectively. The NH_3_ flow was 1000 sccm and TMAl flow was 50 sccm. Unintentional doped GaN (u‐GaN) layer was grown at 1050 °C for 90 min with the NH_3_ flow of 36 slm and TMGa flow of 150 sccm, respectively. Such GaN films can be used directly in the preparation of PDs. The Ni/Au (20/300 nm) interdigitated fingers were deposited via electron beam evaporation. Finger length, spacing and width of MSM structure were 285 µm, 10 µm, and 10 µm, respectively.

For LED, silane (SiH_4_) and bis‐cyclopentadienyl magnesium (Cp_2_Mg) were used for n‐type and p‐type doping, respectively. Then, n‐GaN layer was grown on the above‐mentioned u‐GaN layer at 1045 °C for 40 min with the NH_3_ flow of 3600 sccm and TMGa flow of 100 sccm. 5 periods of InGaN/GaN MQWs were grown at 740 °C/810°C. A p‐GaN layer was subsequently deposited as the cladding layer, following by an annealing process at 720 °C for 10 min under N_2_ ambient to activate the Mg acceptors. After the growth of LED, a Ni/Au stack deposited on the top surface of p‐GaN was used as p‐type contact. Then, to obtain the ohmic contact to p‐GaN, a rapid thermal annealing process was performed in ambient air at 600 °C for 1 min.

### Transfer of GaN‐Based Membrane and Devices

Chemical etching rate was related to the concentration of corrosion solution and flow condition. The peeling of the 2‐inch nitride film occurs within 1 minute using a 49% HF solution. A 2‐inch GaN membrane takes about 2–5 hours using the BOE solution (40% NH_4_F: 49% HF = 1:6). By optimizing the peeling method of PMMA coated on the device surface as a transfer support layer, cracks caused by rapid peeling with high concentrations of HF can be effectively avoided. Meanwhile, excessive corrosion time will not affect the quality of peeling. The wafer was placed horizontally on the wafer carrier. Peeling can proceed when the etching solution penetrates inward from the outside to the center of the wafer, and when the bubbles were evenly distributed in the center. The GaN‐based film was transferred via a transparent thermal‐release tape in Figure 1f. For LED, GaN‐based membrane on the Gr was transferred from the original Si(100) substrate onto copper conductive tape as shown in Figure S9 (Supporting Information). Then take out the soaked sample, lay it flat on the table, cover the front of the sample with a tape, press it three times with a roller, and then lift the tape up. The membrane was transferred to the tape. Additionally, the Gr layer was used as bottom electrode for the vertical LED. The MSM PDs were transferred via a transparent thermal‐release tape because no bottom electrodes were required. Subsequently, the PMMA was dissolved by acetone immersion, and the PD and thermal‐release tape were separated to become independent devices.

### Characterization

The samples were characterized by SEM (Hitachi S‐4800; operating at 3 kV), AFM (D3100, Veeco), Raman spectrum (Horiba, 532 nm laser excitation), HRXRD (BRUKER D8 Discover XRD system with Cu K α radiation, λ ≈ 1.5418 Å), HRTEM (FEI Tecnai F20 TEM, operated at 200 kV). The EL spectrum of blue‐LED was characterized by Integrating sphere (HAAS‐2000). The PL spectra were measured using a detection system equipped with a monochromator and a charge‐coupled device. A continuous He‐Cd laser (325 nm) or a pulsed Nd:YAG laser (355 nm) was employed as an optical excitation source for the PL spectroscopy. The performance of MSM PD was tested at room temperature and in air atmosphere using HSX‐UV300 xenon lamp. The I‐V characteristics and the current‐time (I‐t) curves were measured using a Keithley‐B1500A semiconductor parameter analyzer.

## Conflict of Interest

The authors declare no conflict of interest.

## Author Contributions

Y.G. and K.Z. contributed equally to this work. T.W. and J.S. conceived the idea and designed this work. Y.G., L.W., Y.D. and J.Y. performed the nitride growth experiments and device fabrication under the direction of T.W. and J.W., and Z.T.L. performed the electron microscopy experiments under the direction of P.G. and K.Z., X.G. and W.W contributed to the growth of Gr under the direction of J.S. and Z.L. Y.G. and K.Z. performed the data analysis and wrote the manuscript under the direction of T.W and J.S. All authors contributed to the discussion and analysis of the results.

## Supporting information



Supporting Information

## Data Availability

The data that support the findings of this study are available from the corresponding author upon reasonable request.
