# Supplementary material for: Wafer‐Scale Manufacturing and Crack‐Free Transferring of GaN‐Based Membranes for Flexible Optoelectronics
Source: Adv Sci (Weinh). 2025 Sep 23;12(46):e12193. doi: 10.1002/advs.202512193 (PMC12697778; doi:10.1002/advs.202512193)
Supplement: Supplementary file 1 — Supporting Information [file ADVS-12-e12193-s001.docx]

**Supporting Information**

**Wafer-Scale Manufacturing and Crack-Free Transferring of GaN-Based Membranes for Flexible Optoelectronics**

*Yaqi Gao^#^, Kaixuan Zhou^#^, Zhetong Liu, Lulu Wang, Yiwei Duo, Shenyuan Yang, Jiankun Yang, Xiang Gao, Wenze Wei, Junxi Wang, Peng Gao, Jinmin Li, Zhongfan Liu, Jingyu Sun*, and Tongbo Wei**


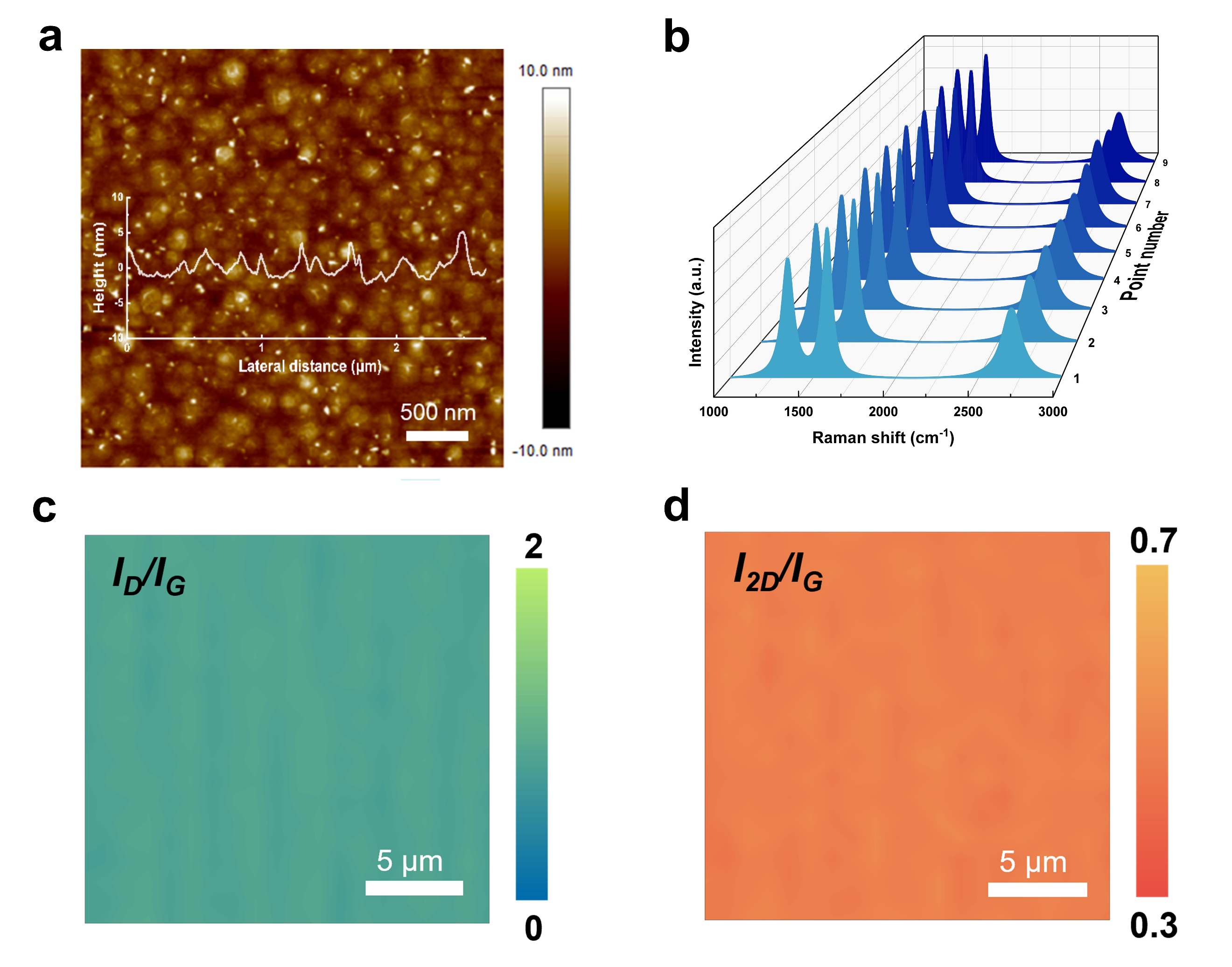


**Figure S1.** Microscopic images and Raman spectra collected from a Gr/SiO_2_/Si wafer. (a) AFM image of Gr grown on Si(100) substrate; (b) Raman spectra collected from the whole area of the Gr/SiO_2_/Si(100) wafer; (c, d) Raman spectra mappings of the Gr/SiO_2_/Si(100) wafer with respect to I_D_/I_G_ (c) and I_2D_/I_G_ (d) ratios.

AFM and Raman spectra results in **Figure S1** both confirm the uniformity of the Gr/SiO_2_/Si(100) wafer. The topography of the as-grown Gr film on the SiO_2_/Si(100) wafer is characterized. Notably, the obtained Gr film displays a remarkably smooth surface, with a RMS roughness value of 1.68 nm (**Figure S1a**). The Raman peaks at 9 positions on the wafer indicate that the uniformity of CVD Gr growth is acceptable (**Figure S1 b**). Statistical analysis of the peak intensity ratios calculated from the line scan reveals average values of I_D_/I_G_ = 0.83±0.02 and I_2D_/I_G_ = 0.47±0.02, respectively, indicative of the high homogeneity of the Gr layer on SiO_2_/Si(100). Additionally, mappings of I_D_/I_G_ and I_2D_/I_G_ for the Gr/SiO_2_/Si(100) wafer are performed, further confirming the nano-scale uniformity of the as-grown sample (**Figures S1c-1d**). As the number of epitaxial Gr layers increases, the frequency shift of the Raman G towards lower wavenumber.


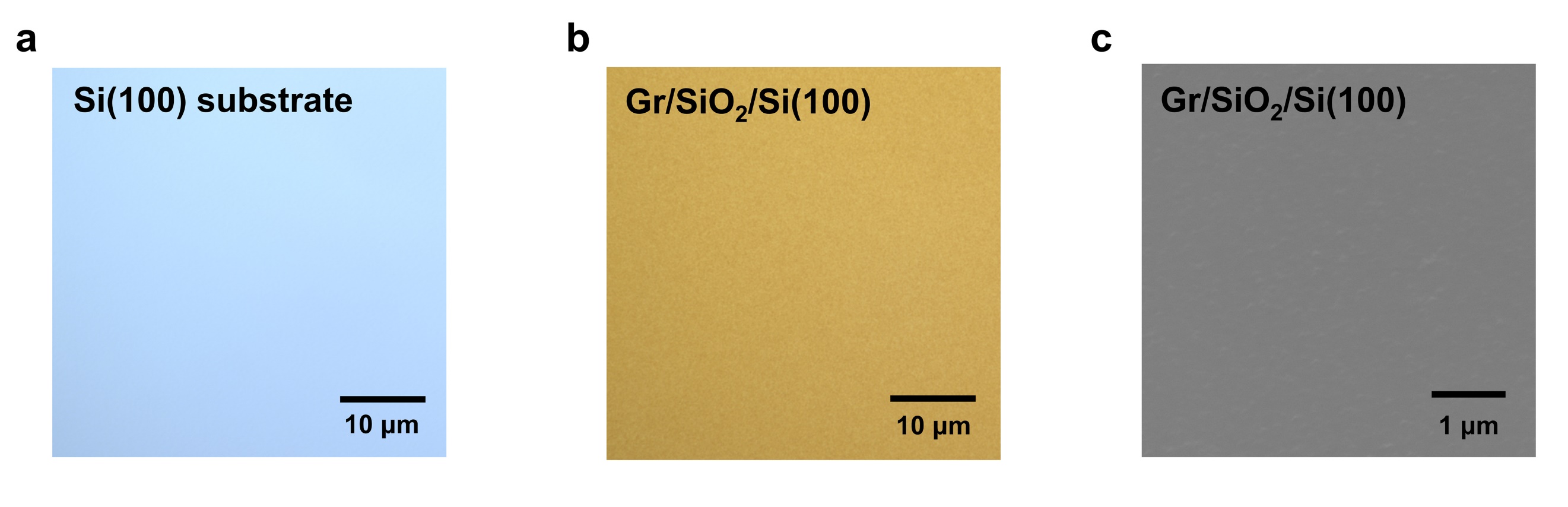


**Figure S2.** Optical microscopy (OM) images of (a) Si(100) substrate; (b) Gr/SiO_2_/Si(100) wafer; (c) SEM image of Gr/SiO_2_/Si(100) wafer. These images indicate the Gr/SiO_2_/Si(100) wafer has good coverage. There are no noticeable impurity particles, which provides good uniformity for subsequent epitaxial nitrides. Microscopy images of Si(100) substrate and Gr/SiO_2_/Si(100) wafer were shown in **Figure S2 (a-c)**, which indicate the Gr/SiO_2_/Si(100) wafer has good coverage at the microscale.


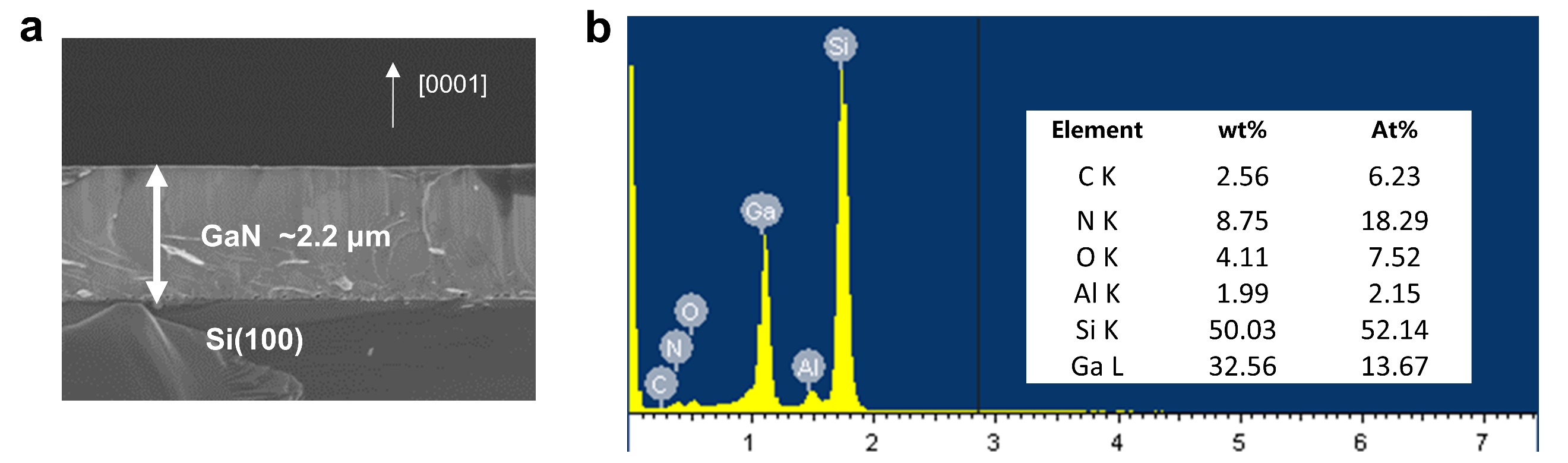


**Figure S3.** SEM images (a) and EDS spectrum (b) of cross-section of GaN film. The GaN film thickness is about 2.2 μm. The statistical results of Ga, Al, C, Si, O, and N elements are presented, which also indicates that graphene still exists after the growth of GaN epitaxial layer.


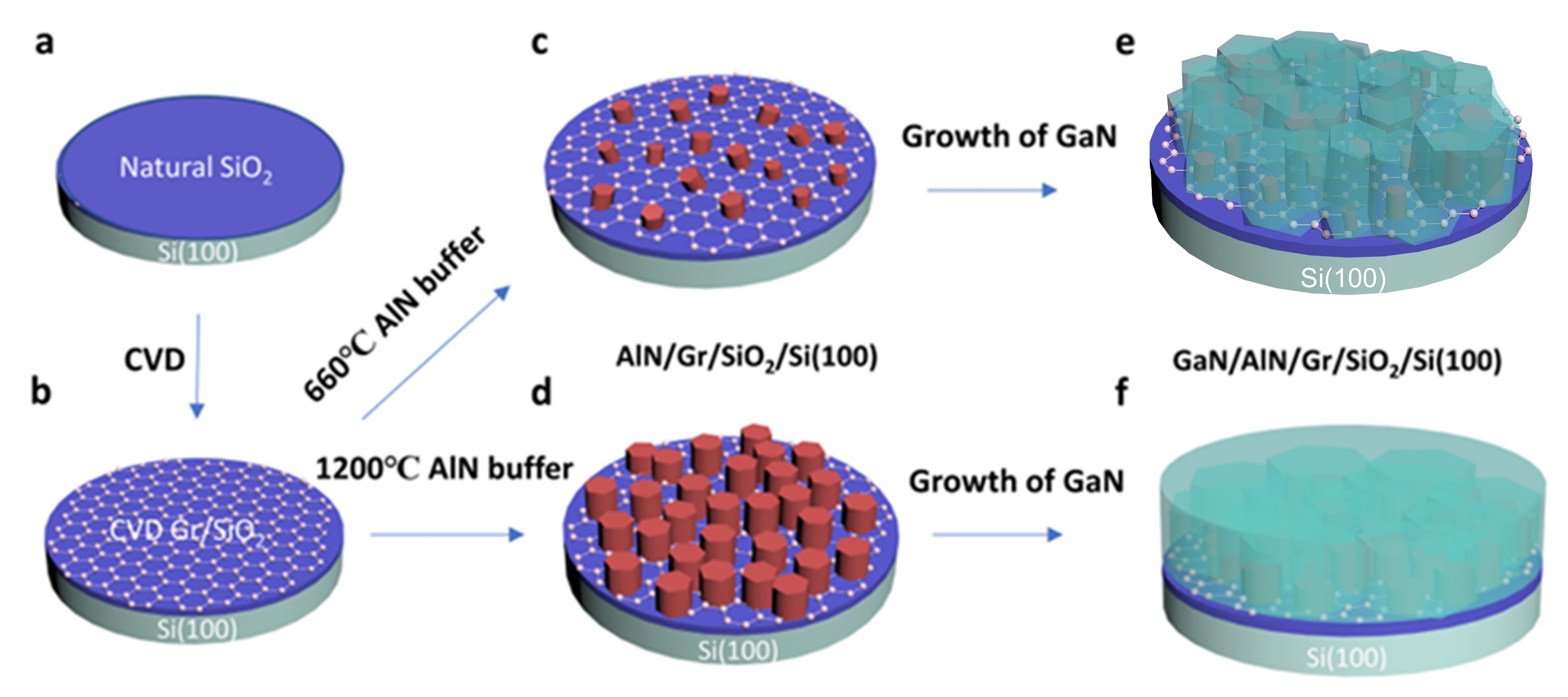


**Figure S4.** Schematic diagram of epitaxial growth of GaN films. (a) CMOS-compatible SiO_2_/Si(100) substrate; (b) Growth of CVD single-crystalline Gr on a Si(100) substrate; (c) AlN nucleation islands grown on Gr/SiO_2_/Si(100) at 660 ℃; (d) AlN nucleation islands grown on Gr/SiO_2_/Si(100) at 1200 ℃; (e) Polycrystalline GaN epilayer grown on LT-AlN/Gr/SiO_2_/Si(100) substrate; (f) Nearly single-crystalline GaN epilayer grown on HT-AlN/Gr/SiO_2_/Si(100) substrate.


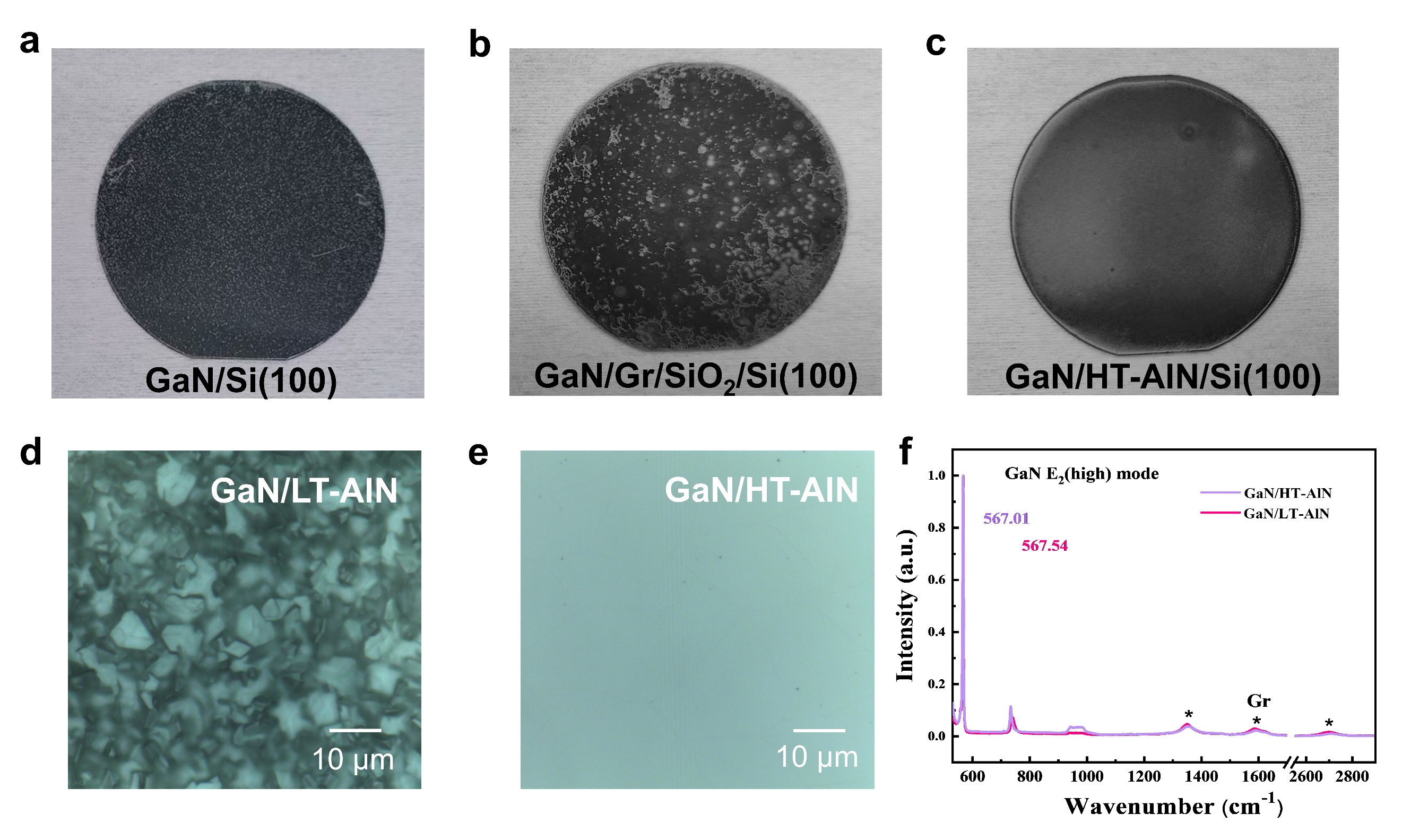


**Figure S5.** (a) Photograph of GaN/Si(100); (b) Photograph of GaN/Gr/SiO_2_/Si(100); (c) Photograph of GaN/HT-AlN/Si(100); OM images of GaN/LT-AlN (d) and GaN/HT-AlN (e); (f) Raman spectra results of GaN/LT-AlN and GaN/HT-AlN;


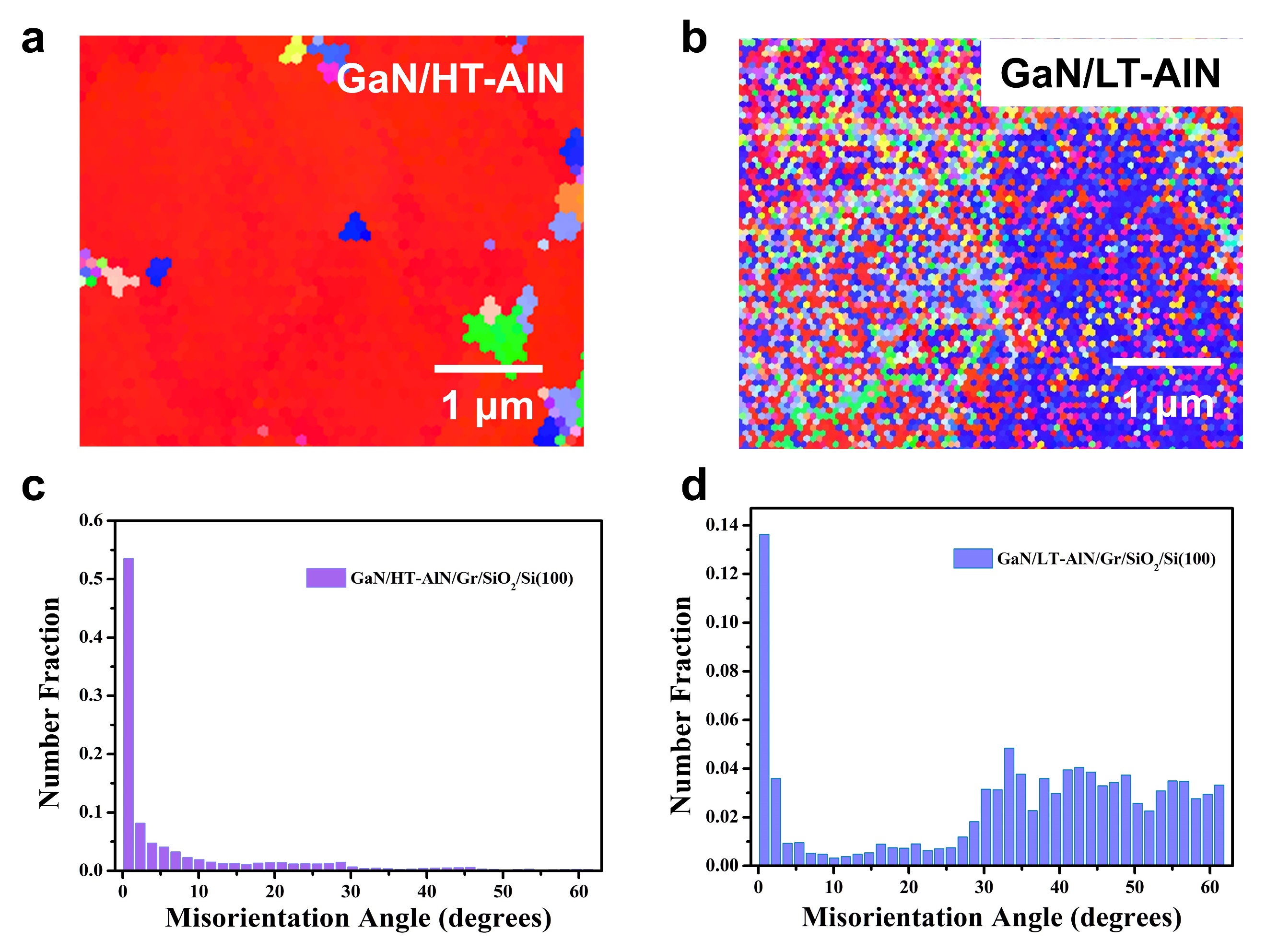


**Figure S6.** EBSD IPF images of GaN/HT-AlN (a) and GaN/LT-AlN (b); Statistics of misorientation angles for GaN/HT-AlN (c) and GaN/LT-AlN (d). HT-AlN nucleation layer avoides the formation of high angle grain boundaries with disordered atomic arrangement.


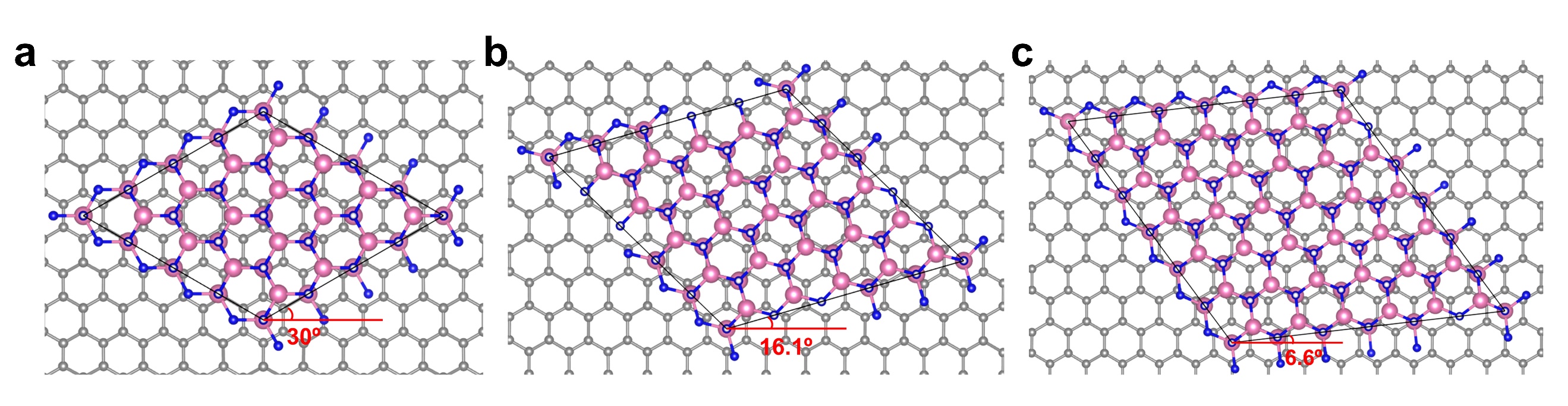


**Figure S7.** Three lattice-matched epitaxial relationships between GaN and Gr system. The DFT results indicate the GaN domains with several twist angles could coexist.


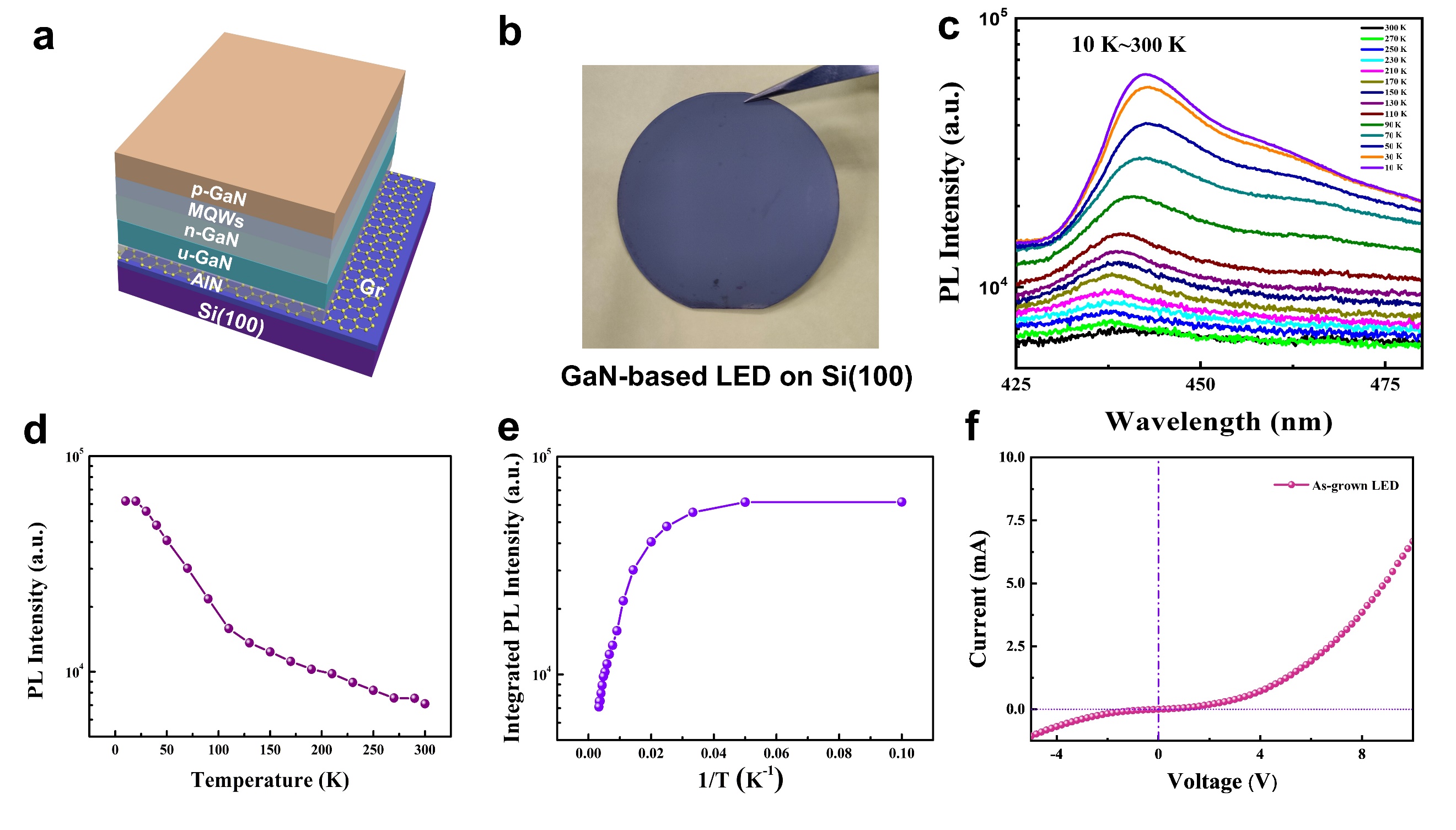


**Figure S8.** (a) Schematic diagram of LED structure; (b) Photograph of 2-inch LED film; (e) Temperature dependence PL spectra; (d) The normalized integrated PL intensity as a function of temperature; (e) Temperature dependence of normalized PL intensity in the form of an Arrhenius plot, log(I/T) versus 1/T, in which I is integrated PL intensity, T is temperature of PL measurement; (f) I-V curves of as-grown GaN-based LED.


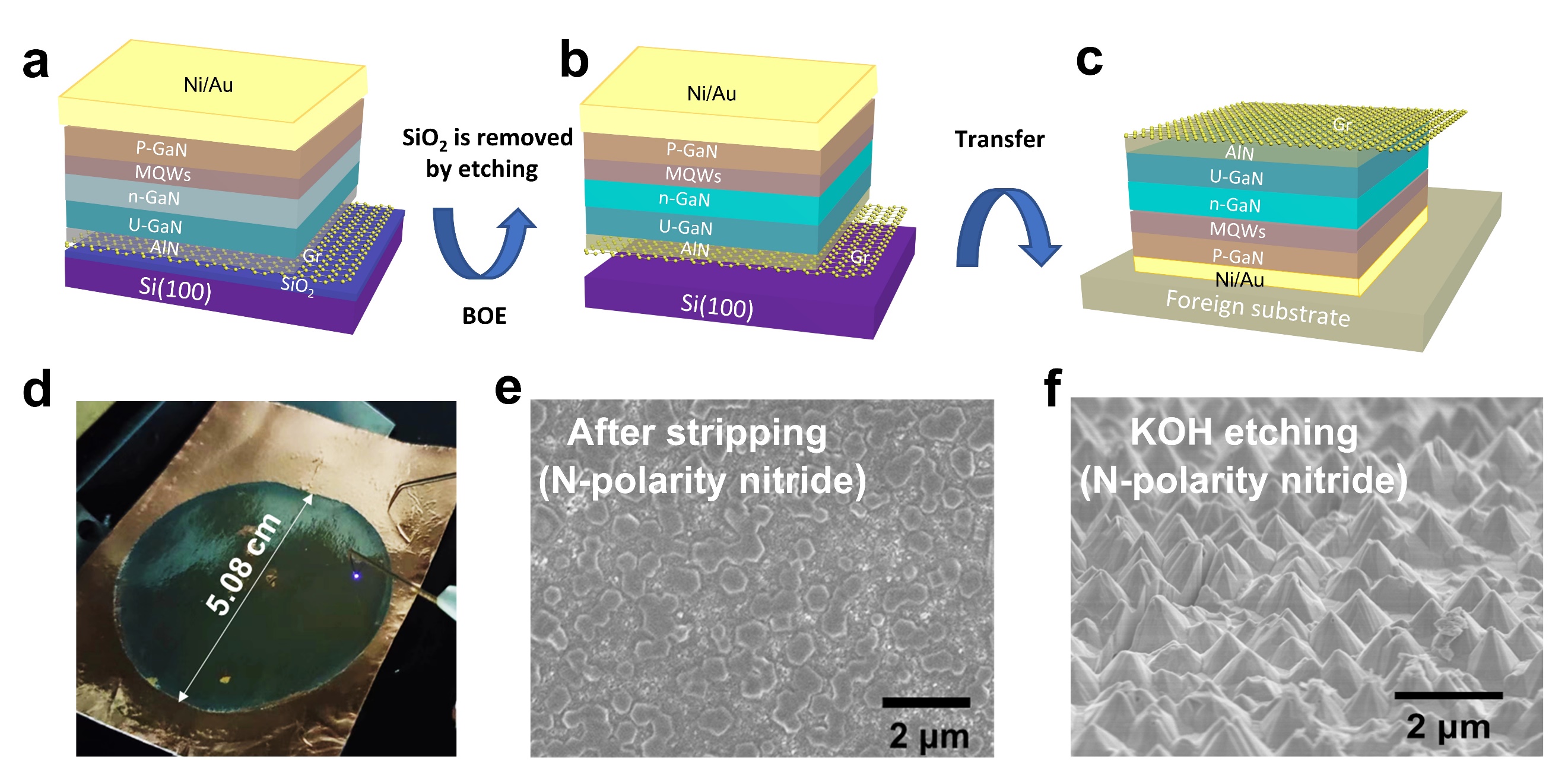


**Figure S9.** (a-c) Schematic diagram of LED transferred to foreign substrate; (d) Photograph of the vertical structure LED membrane; (e) The SEM image of membrane surface with LT-buffer layer after stripping; (f) The SEM images of N-polarity nitride after KOH etching.

As shown in Figure S9(a-c), we transfer the Ni/Au/LED membrane to the copper tape. First, lay the soaked sample flat on a tabletop, cover the front surface of the sample with copper tape, press three times using a roller, and then lift the copper tape upwards. The SEM images illustrate that the initial nucleation density directly affects the morphology and quality of the backside of the nitride after stripping. The importance of the growth temperature and time of the AlN nucleation layer is confirmed. These results also demonstrated that the N-polarity face of nitride film after etching SiO_2_ is sufficiently flat to be used for vertically structured LED.

And the N-polarity face nitride film is prone to KOH etching and exhibits rough morphology. Through this etching process, the light extraction efficiency (LEE) of LEDs may be greatly improved. Due to the selective orientation of crystal planes, after the N-face GaN is etched by KOH solution, the angle between the hexagonal pyramid surface and the (0001) surface is 28°, and the hexagonal pyramid surface exhibits the (10-11) plane.^[1-2]^ As the etching time increases, the grain size increases to a certain extent before stopping, while the grain density on the surface continuously decreases, until the grain size begins to increase again. The increase in surface roughness and grain size due to prolonged etching time can effectively enhance the output optical intensity of the chip. However, when the grain size remains essentially unchanged while the density continues to increase, the probability of secondary absorption of light between grains increases, which is detrimental to the optical output of the chip. On the other hand, excessive surface roughness after etching is unfavorable for the fabrication of the n-electrode, and increased etching time can negatively impact the transfer substrate. Therefore, it is necessary to find an appropriate etching time and etching solution temperature while enhancing LEE. The LEDs in our main text do not use this method, as corrosion uniformity issues can lead to more variation, which makes the analysis difficult. The device preparation in the main text needs to consider the reaction of the tape in the KOH solution to avoid LED rupture caused by its expansion and deformation.


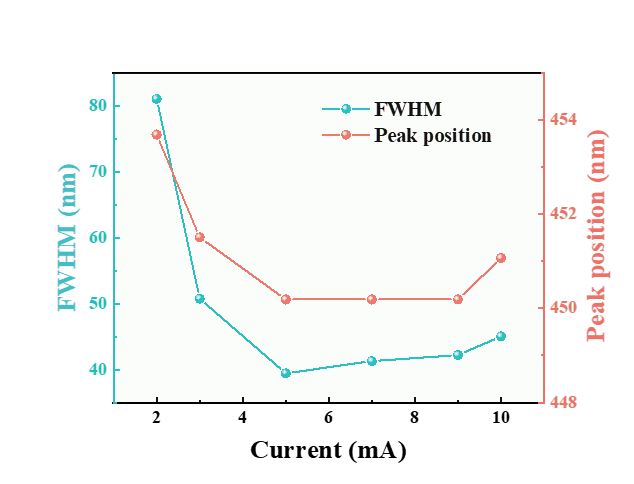


**Figure S10.** The peak position and FWHM values as a function of the applied current for flexible GaN-based LED.


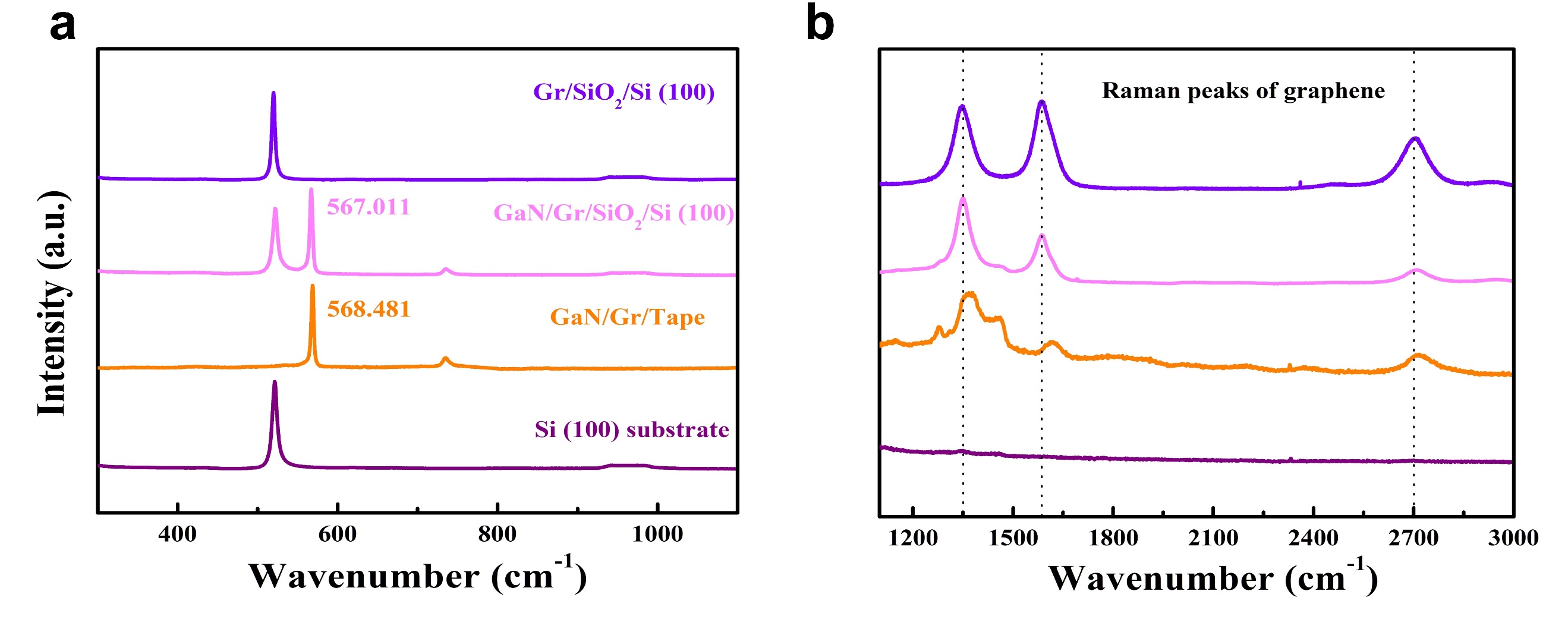


**Figure S11.** Raman spectra of nitride film (a) and Gr (b) before and after the transfer. The Raman results further demonstrated the successful implementation of epilayer peeling, and Gr exists on the N-polarity face of epilayer after peeling.


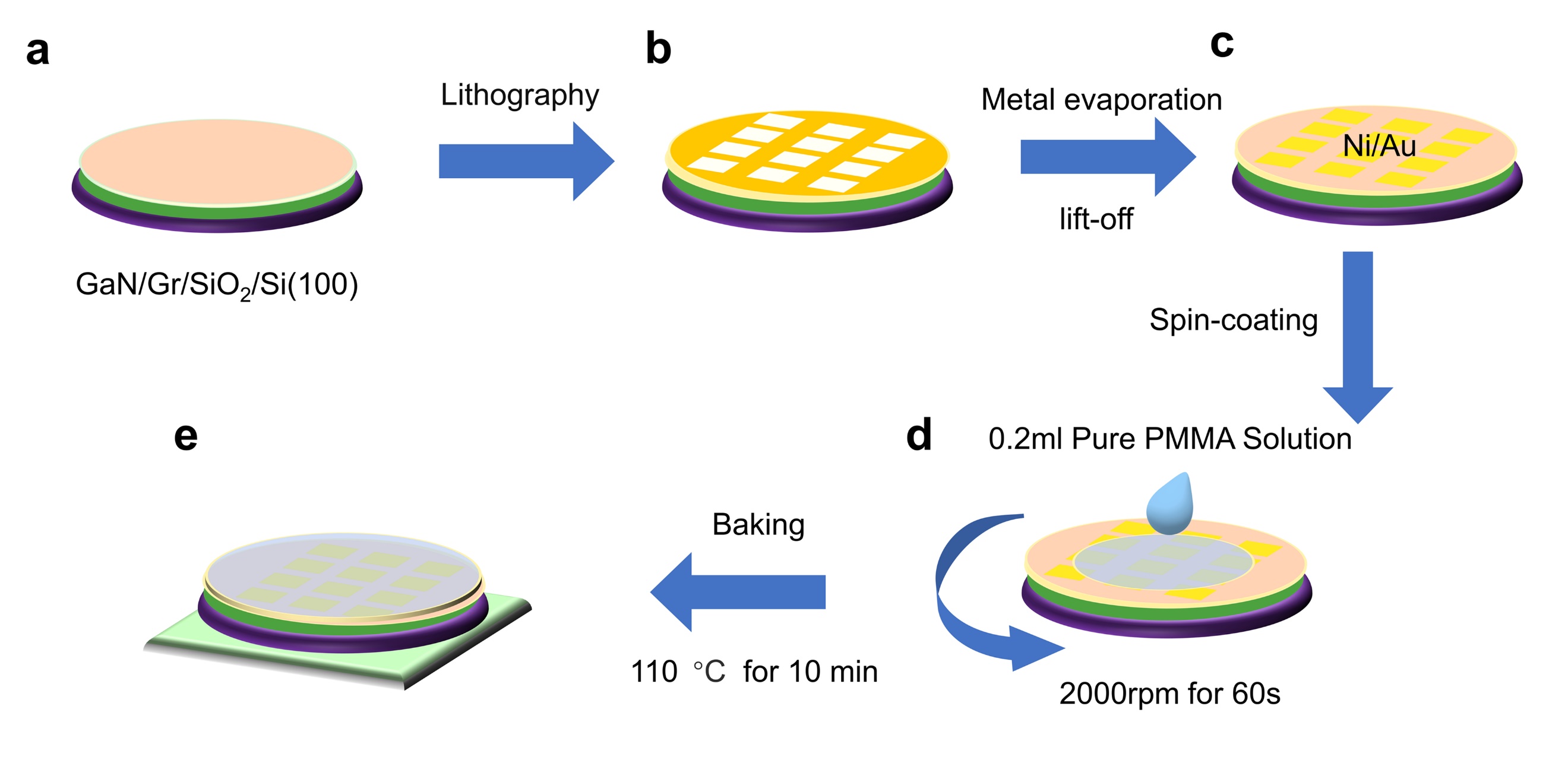


**Figure S12.** The Schematic diagram of preparation and transfer process for GaN-based MSM PD: (a) GaN epitaxial on a Gr/SiO_2_/Si(100) substrate; (b) The patterns are formed on the photoresist by lithography; (c) Metal evaporation for the deposition of Ni/Au stack and lift-off process for pattern transfer; (d) GaN-based MSM PD was spin-coated with the 0.2 ml pure PMMA solution at 2000 rpm for 60s; (e) The coated films were baked at 110 °C for 10 min to protect the electrodes from damage during the exfoliation of the GaN-based MSM PD membrane.


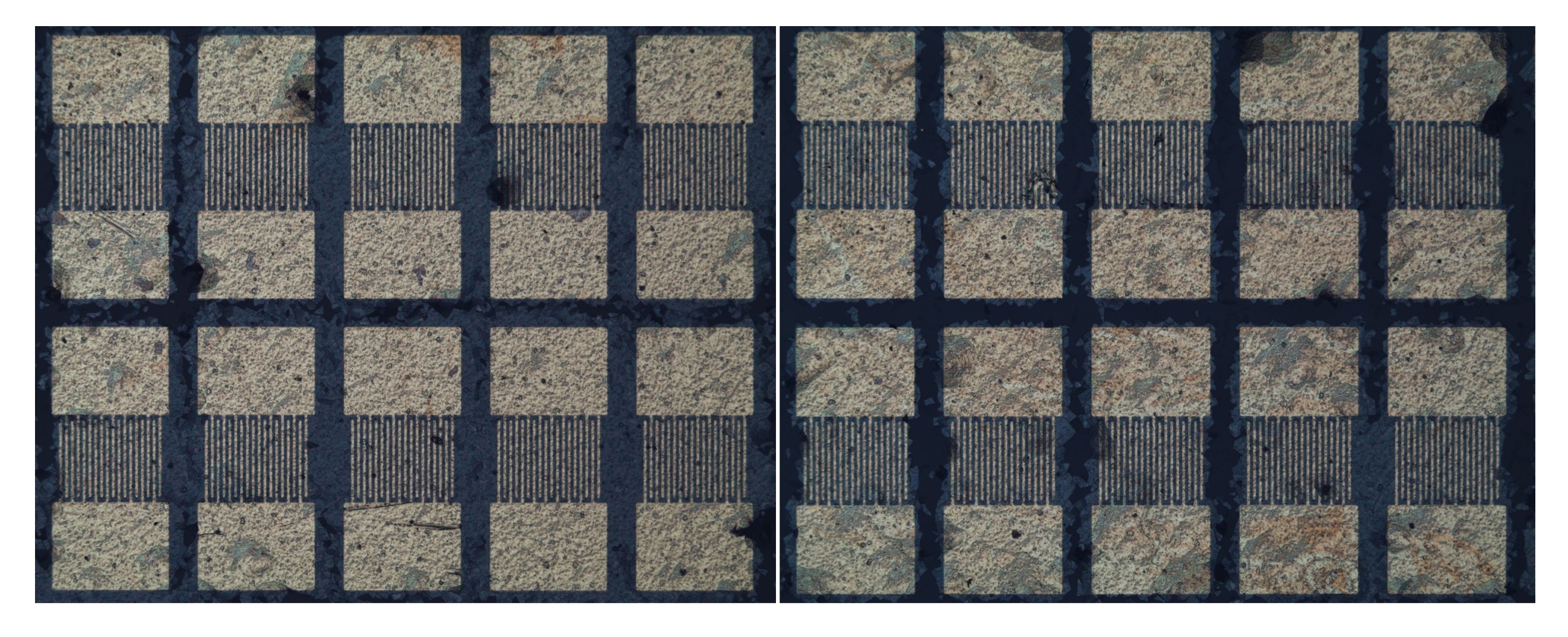


**Figure S13.** OM images of the PD arrays with low damage after dissolving the PMMA.


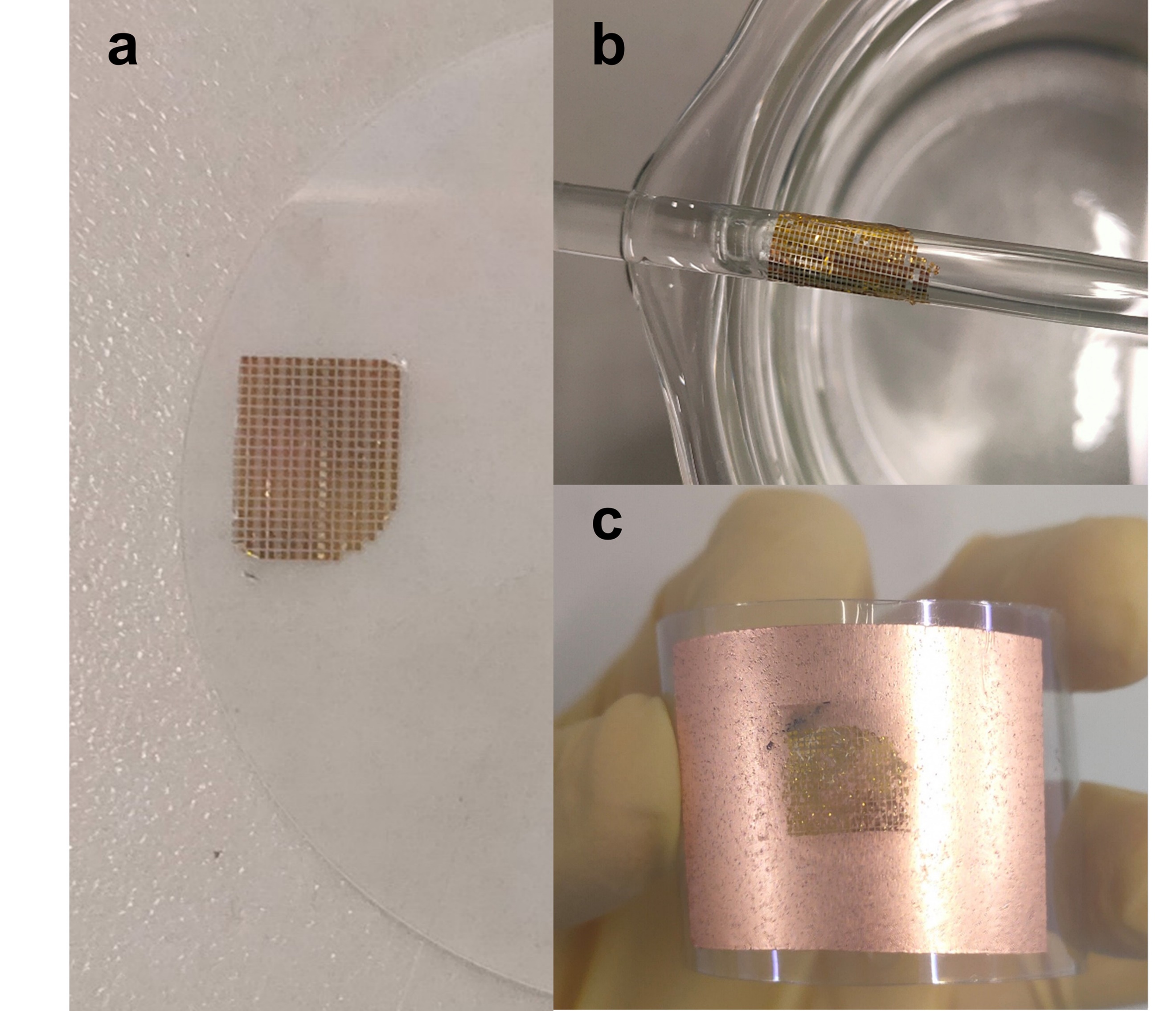


**Figure S14.** The flexible GaN PDs with MSM electrodes can be transferred to any substrate, as shown in (a) sapphire, (b) glass rod, (c) copper foil. The membrane has good flexibility to achieve the function of UV detection.


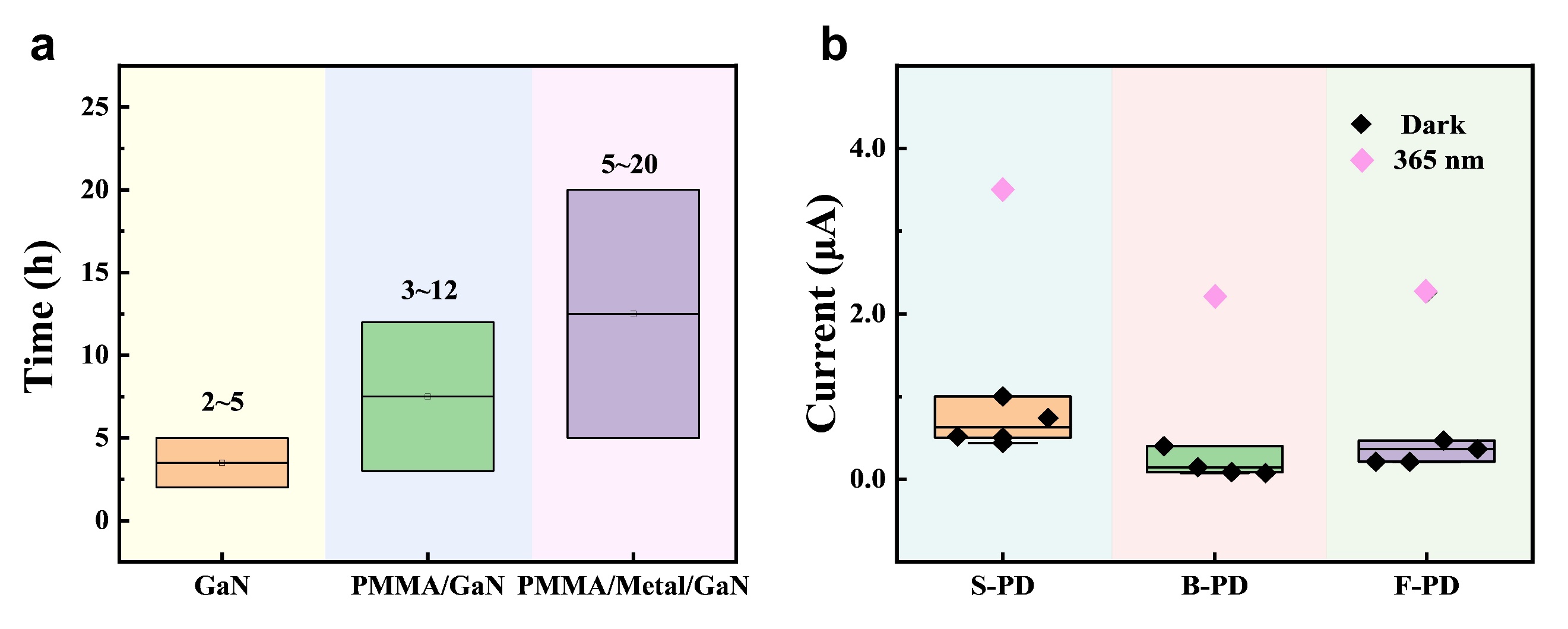


**Figure S15.** (a) Etching time range of films coated with different materials; (b) The current characteristic of the MSM PD. (S-PD: the PD before peeling; B-PD: the PD still on Si(100) substrate after the etching of SiO_2_; F-PD: the PD was transferred to a foreign substrate). It is noted that the time for HF etching resistance varies depending on the substance coated on the GaN surface. During the entire transfer process, the dark current of the MSM PD did not significantly increase and its performance remained good.


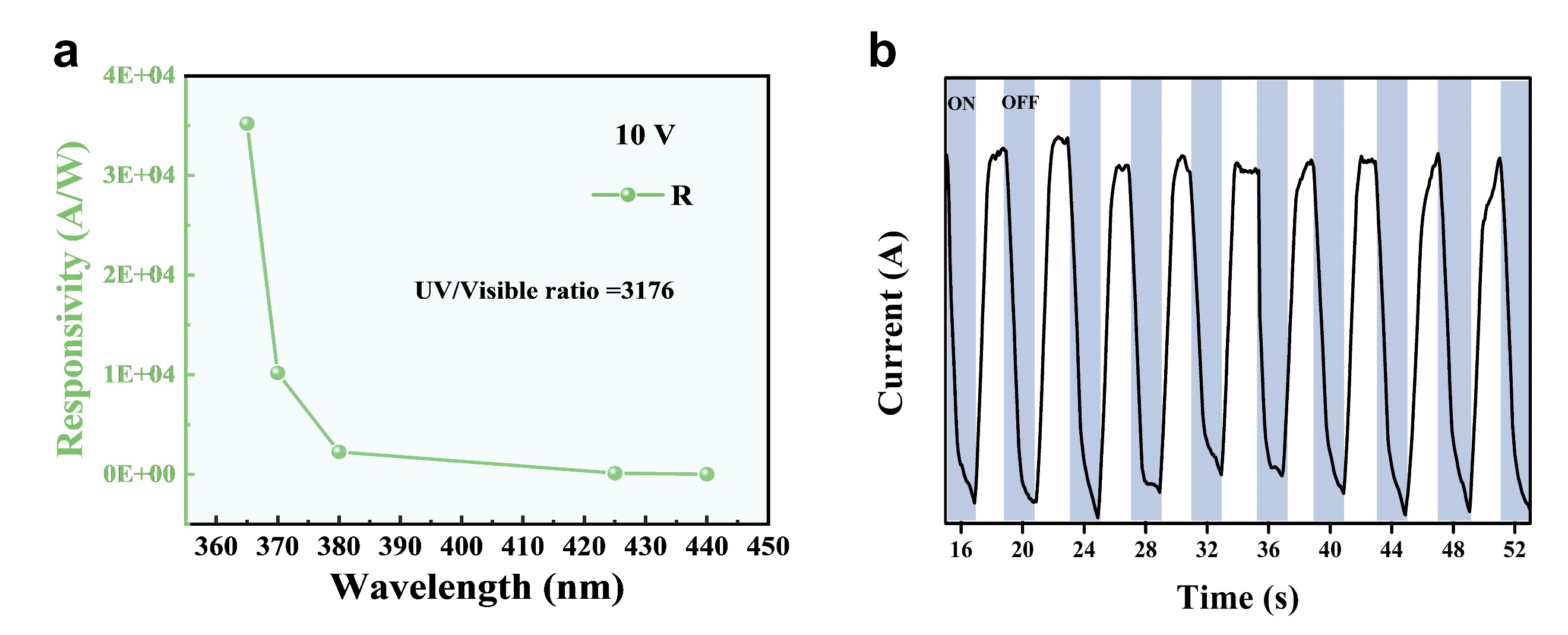


**Figure S16.** (a) The wavelength-dependent responsivity measured in the range of 365~440 nm; (b) The I-T curve of flexible GaN-based PD MSM structure. Detection in multiple on-off processes has proven the stability of the flexible MSM PD.


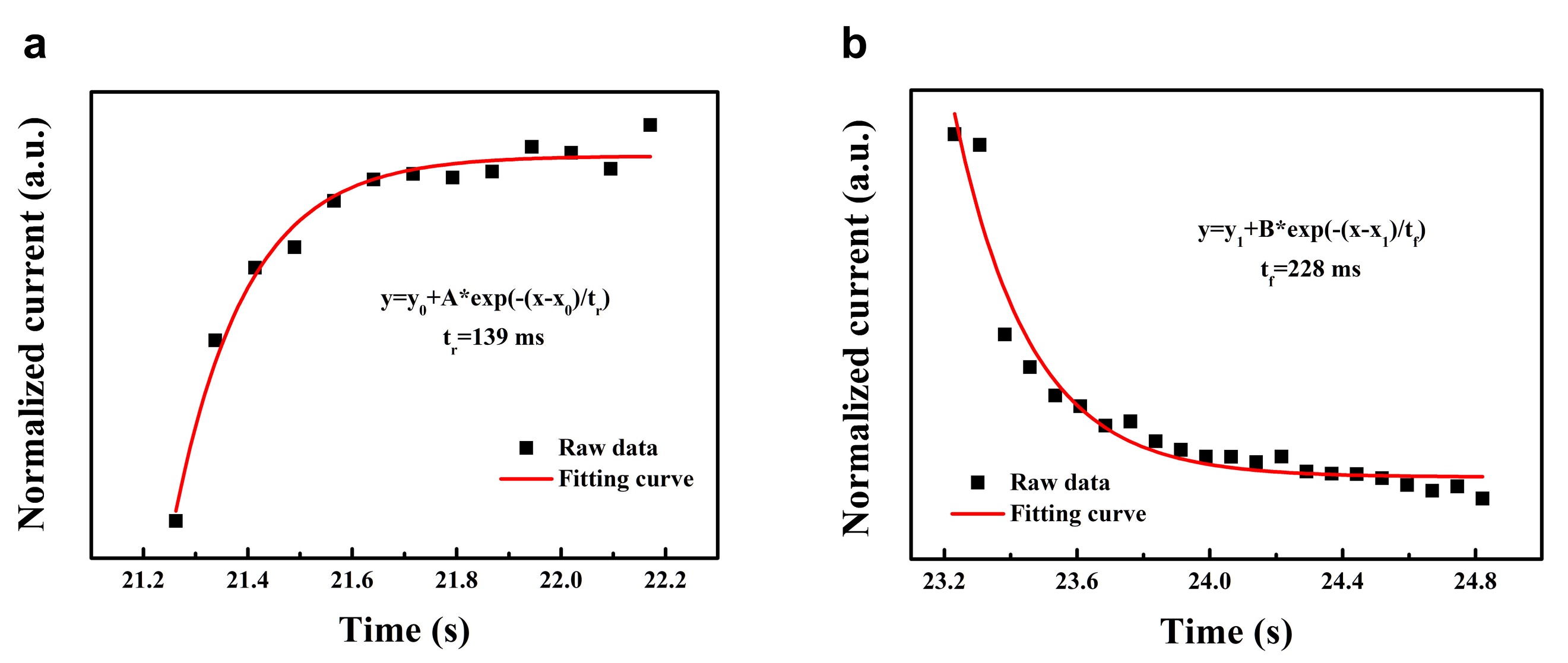


**Figure S17.** The fitting curves of response time for flexible GaN-based PD MSM structure: (a) Rise time; (b) Fall time. Single exponential equation is used to evaluate response time. In multiple 2 s on/off tests using the ordinary xenon lamp, the rise and fall time are 139 and 228 ms, respectively.


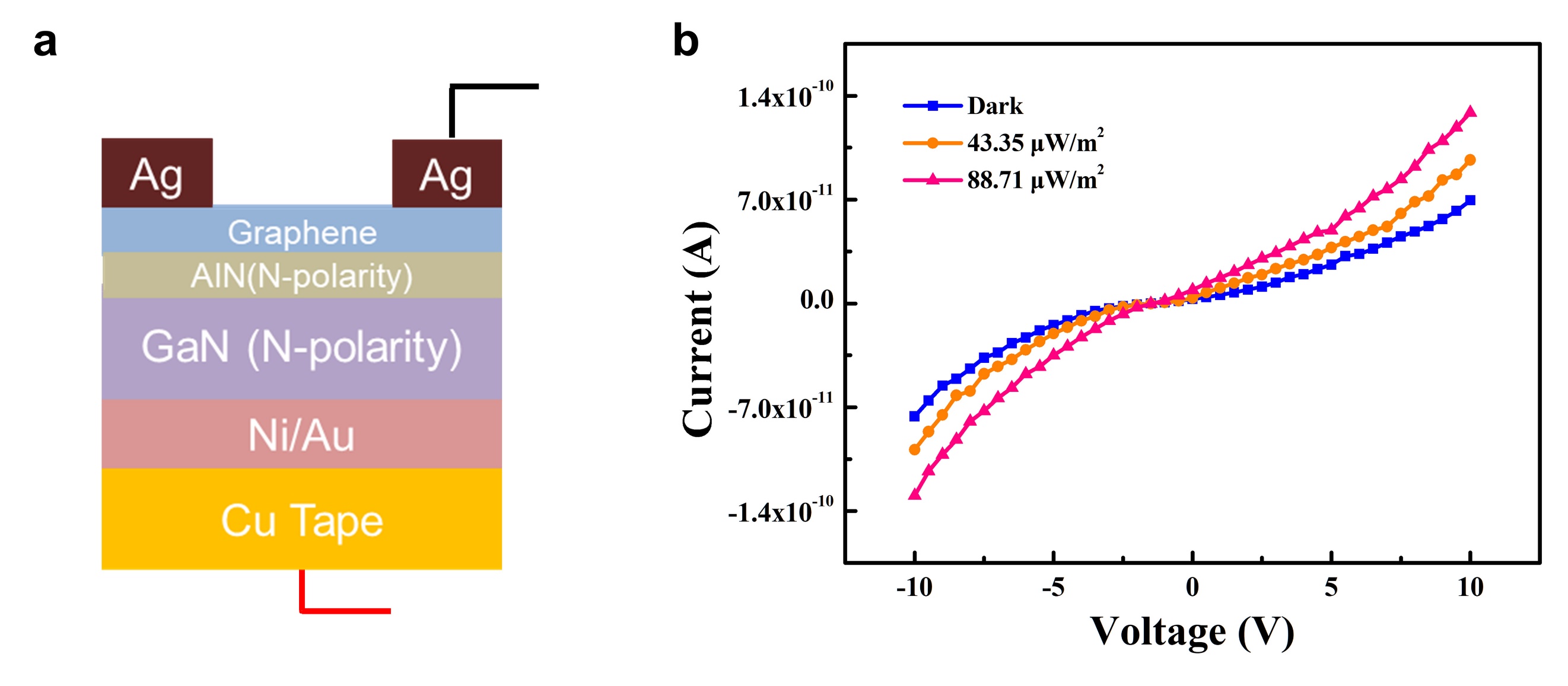


**Figure S18.** The diagrammatic sketch (a) and I-V curves (b) of flexible vertical conductive structure PD. The dark current and photocurrent of the PD are both low, resulting in poor photo-response performance. The dark current is 69.53 pA at 10 V, the responsivity is 0.9 mA/W, and the specific detectivity is 5.39×10^7^ Jones. Compared to PD with a vertical structure made of the GaN membrane on Gr/SiO_2_/Si(100), the transverse conductive MSM PD has excellent performance as mentioned in the main text.

**Table S1.** Summary of the recent examples of GaN grown on amorphous substrates. Compared to previous reports, the quality of GaN has been improved, and flexible vertical LED and flexible MSM PD with good performance are presented in our work.

| **Substrate** | **Epitaxial equipment** | **Epitaxial layer** | **FWHM（0002）（°）** | **Device** | **Year** | **Ref.** |
| --- | --- | --- | --- | --- | --- | --- |
| Gr/SiO_2_/Si | MOCVD | GaN/ZnO nanorods | -- | Flexible LED | 2011 | [3] |
| Gr/SiO_2_/Si | MOCVD | GaN film/ZnO nanowires | 0.8 | LED | 2012 | [4] |
| Gr/SiO_2_/Si(100) | MOCVD | GaN micro-rod | 2.5 | Flexible LED | 2014 | [5] |
| Multilayer Gr/SiO_2_ | Pulsed Sputtering Deposition | GaN film | 0.617 | No | 2014 | [6] |
| Single-crystalline Gr/PVD AlN/ SiO_2_/Si(100) | MOCVD | GaN film | 0.59 | No | 2022 | [7] |
| Gr/Glass | MOCVD | GaN film | 1.2 | Flexible LED | 2021 | [8] |
| Gr/Glass | MOCVD | GaN film | 1.19 | PD | 2022 | [9] |
| Gr/Glass | MOCVD | GaN film | Polycrystalline | No | 2023 | [10] |
| BN/Glass | MOCVD | GaN film | 2.37 | No | 2017 | [11] |
| WS_2_-glass | MOCVD | GaN film | 1.6 | LED | 2022 | [12] |
| WS_2_-glass | MOCVD | GaN film | 1.5 | No | 2023 | [10] |
| Gr/SiO_2_/Si(100) | MOCVD | GaN film | 0.56 | Flexible LED&PD | 2025 | This work |

**REFERENCES**

[1] Fan J M, Wang L C, Liu Z Q. The Influence of Surface Roughening on GaN Based Vertical-Electrodes LEDs. *Journal of Optoelectronics·Laser,* **2009**, 8: 994-996.

[2] Zheng Q H, Liu B L, Zhang B P. Simulation of the Enhancement of Photon Extraction Efficiency of GaN-Based LED via Surface Roughening. *Chinese Journal of Electron Devices*, **2008**, 31, 4.

[3] Lee C-H, Kim Y-J, Hong Y J, Jeon S-R, Bae S, Hong B H, Yi G-C. Flexible Inorganic Nanostructure Light-Emitting Diodes Fabricated on Graphene Films. *Advanced Materials,* **2011**, 23, 4614-4619.

[4] Chung K, In Park S, Baek H, Chung J-S, Yi G-C, High-Quality GaN Films Grown on Chemical Vapor-Deposited Graphene Films. *NPG Asia Materials,* **2012**, 4, e24-e24.

[5] Chung K, Beak H, Tchoe Y, Oh H, Yoo H, Kim M, Yi G-C, Growth and Characterizations of GaN Micro-Rods on Graphene Films for Flexible Light Emitting Diodes. *APL Materials,* **2014**, 2, 092512.

[6] Shon J W, Ohta J, Ueno K, Kobayashi A, Fujioka H, Structural Properties of GaN Films Grown on Multilayer Graphene Films by Pulsed Sputtering. *Applied Physics Express*, **2014**, 7, 085502.

[7] Liu D, Hu L, Yang X, Zhang Z, Yu H, Zheng F, Feng Y, Wei J, Cai Z, Chen Z, Ma C, Xu F, Wang X, Ge W, Liu K, Huang B, Shen B, Polarization-Driven-Orientation Selective Growth of Single-Crystalline III-Nitride Semiconductors on Arbitrary Substrates. *Advanced Functional Materials,* **2022**, 32. 2113211.

[8] Ren F, Liu B, Chen Z, Yin Y, Sun J, Zhang S, Jiang B, Liu B, Liu Z, Wang J, Liang M, Yuan G, Yan J, Wei T, Yi X, Wang J, Zhang Y, Li J, Gao P, Liu Z, Liu Z, Van Der Waals Epitaxy of Nearly Single-Crystalline Nitride Films on Amorphous Graphene-Glass Wafer. *Science Advances*, **2021**, 7, eabf5011.

[9] Jiang B, Liang D, Sun Z, Ci H, Liu B, Gao Y, Shan J, Yang X, Rümmeli M H, Wang J, Wei T, Sun J, Liu Z, Toward Direct Growth of Ultra‐Flat Graphene. *Advanced Functional Materials,* **2022**, 32, 2200428.

[10] Chen Q, Yang K, Shi B, Yi X, Wang J, Li J, Liu Z, Principles for 2D-Material-Assisted Nitrides Epitaxial Growth*. Advanced Materials,* **2023**, 35, e2211075.

[11] Chung K, Oh H, Jo J, Lee K, Kim M, Yi G-C, Transferable Single-Crystal GaN Thin Films Grown on Chemical Vapor-Deposited Hexagonal BN Sheets. *NPG Asia Materials,* **2017**, 9, e410-e410.

[12] Yin Y, Liu B, Chen Q, Chen Z, Ren F, Zhang S, Liu Z, Wang R, Liang M, Yan J, Sun J, Yi X, Wei T, Wang J, Li J, Liu Z, Gao P, Liu Z, Continuous Single-Crystalline GaN Film Grown on WS_2_-Glass Wafer. *Small.* **2022**, 18, 2202529.
